# Multi‐material Electrohydrodynamic Printing of Bioelectronics with Sub‐Microscale 3D Gold Pillars for In Vitro Extra‐ and Intra‐Cellular Electrophysiological Recordings

**DOI:** 10.1002/advs.202407969

**Published:** 2025-01-10

**Authors:** Bingsong Gu, Qihang Ma, Jiaxin Li, Wangkai Xu, Yuke Xie, Peng Lu, Kun Yu, Ziyao Huo, Xiao Li, Jianhua Peng, Yong Jiang, Dichen Li, Jiankang He

**Affiliations:** ^1^ State Key Laboratory for Manufacturing Systems Engineering Xi'an Jiaotong University Xi'an 710049 P. R. China; ^2^ National Medical Products Administration (NMPA) Key Laboratory for Research and Evaluation of Additive Manufacturing Medical Devices Xi'an Jiaotong University Xi'an 710049 P. R. China; ^3^ State Industry‐Education Integration Center for Medical Innovations Xi'an Jiaotong University Xi'an 710049 P. R. China; ^4^ Laboratory of Neurological Diseases and Brain Function The Affiliated Hospital of Southwest Medical University Luzhou 64600 P. R. China

**Keywords:** 3D gold pillars, bioelectronics, electrohydrodynamic printing, electrophysiological recording, multi‐material printing

## Abstract

Micro/nanoscale 3D bioelectrodes gain increasing interest for electrophysiological recording of electroactive cells. Although 3D printing has shown promise to flexibly fabricate 3D bioelectronics compared with conventional microfabrication, relatively‐low resolution limits the printed bioelectrode for high‐quality signal monitoring. Here, a novel multi‐material electrohydrodynamic printing (EHDP) strategy is proposed to fabricate bioelectronics with sub‐microscale 3D gold pillars for in vitro electrophysiological recordings. EHDP is employed to fabricate conductive circuits for signal transmission, which are passivated by polyimide via extrusion‐based printing. Laser‐assisted EHDP is developed to produce 3D gold pillars featuring a diameter of 0.64 ± 0.04 µm. The 3D gold pillars demonstrate stable conductivity under the cell‐culture environment. Living cells can conformally grow onto these sub‐microscale 3D pillars with a height below 5 µm, which facilitates the highly‐sensitive recording of extracellular signals with amplitudes <15 µV. The 3D pillars can apply electroporation currents to reversibly open the cellular membrane for intracellular recording, facilitating the measurement of subtle cellular electrophysiological activities. As a proof‐of‐concept demonstration, fully‐printed chips with multiple culturing chambers and sensing bioelectronics are fabricated for zone‐specific electrophysiological recording in drug testing. The proposed multi‐material EHDP strategy enables rapid prototyping of organ‐on‐a‐chip systems with 3D bioelectronics for high‐quality electrophysiological recordings.

## Graphical abstract

A novel multi‐material EHDP strategy is proposed to produce bioelectronics with sub‐microscale 3D gold pillars for in vitro electrophysiological recordings. The printed bioelectronics demonstrate stable conductivity, and the 3D gold pillars enabled intracellular access, facilitating the acquisition of high‐quality electrophysiological signals from cells. This strategy demonstrates promise for rapid prototyping of organ‐on‐a‐chip systems with 3D bioelectronics.

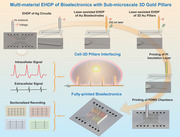

## 1 Introduction

Electrophysiological signals play a vital role in the function of electrogenic tissues, such as the cardiac electrical‐mechanical synchronized contraction and the rapid neuronal network communication.^[^ ^1^, ^2^ ^]^ The generation of electrophysiological signals mainly relies on the minute ionic flows across the cellular membrane.^[^ ^3^ ^]^ High‐quality electrophysiological recording provides an effective way to measure the feature phase of the electrophysiological signals, reflecting the states of the ionic flows.^[^ ^4^ ^]^ For example, the patch‐clamp, which has been widely considered as a gold standard in the field of electrophysiological recordings, allows both the amplitude and shape of the electrophysiological signals to be recorded faithfully with high signal‐to‐noise ratios but is limited by low throughput and high labor costs.^[^ ^5^ ^]^ The advance of bioelectronics enables researchers to fabricate micro/nanoscale bioelectrodes that can support cellular adhesion and growth to form tight cell‐electrode interfaces, which provides a powerful tool for non‐invasive, long‐term, and multiplexed electrophysiological recording in vitro and in vivo.^[^ ^6^, ^7^, ^8^ ^]^ Nevertheless, conventional bioelectrodes with planar structures suffer from low signal quality and resolution, which makes them unable to accurately measure the minute electrophysiological signals required for studying the cellular activities of ion channels.^[^ ^9^, ^10^, ^11^ ^]^ Recent efforts have been made to integrate micro/nanoscale 3D structures with conventional planar bioelectrodes for high‐quality electrophysiological measurement. This integration has shown promising results in enabling the bioelectrode to conform with living cells and access deep intra‐cellular regions, thus facilitating the acquisition of high signal‐to‐noise ratio extra‐ and intra‐cellular electrophysiological signals.^[^ ^12^, ^13^, ^14^, ^15^, ^16^ ^]^

To fabricate bioelectronics with tiny 3D bioelectrodes for electrophysiological recordings, both in‐organic and organic electronic materials have been employed, and different fabrication processes were developed including micro/nanofabrication and 3D printing techniques.^[^ ^17^, ^18^, ^19^ ^]^ Conventionally, micro/nanoscale 3D structures were prepared on silicon‐based substrates via multi‐step physical/chemical depositing and etching processes, and subsequently coated with biocompatible metals (e.g., gold, platinum, titanium) to act as conductive 3D bioelectrodes.^[^ ^20^, ^21^, ^22^, ^23^, ^24^, ^25^, ^26^ ^]^ For example, Robinson et al. further developed pillar‐shaped bioelectrodes with titanium/gold coated tips with the size of 150 nm in diameter and 3 µm in height via micro/nanofabrication techniques. The sub‐microscale 3D bioelectrodes enabled a seamless conformation with rat cortical neuron to penetrate the cellular membrane and realized the acquisition of intra‐cellular signals with amplitudes over 10 mV, demonstrating signal quality comparable to the standard patch clamp technique.^[^ ^27^ ^]^ More recently, organic materials have been applied to produce the 3D bioelectronics by microfabrication techniques. For example, Liu et al. fabricated poly(3,4‐ethyl‐enedioxythiophene): poly(styrenesulfonate) (PEDOT: PSS) hydrogel‐based micropillar bioelectrodes taking advantage of the photolithography, electron beam lithography and reactive‐ion etching techniques. The produced 3D bioelectrodes showed a diameter of 3 µm and enabled to record the extracellular electrophysiological signals from HL‐1 cardiac cells. In addition, the soft nature of the PEDOT:PSS hydrogel enabled to reduce the mechanical mismatch at the electrode‐cell interface.^[^ ^28^ ^]^ Nevertheless, the highly specialized and complicated microfabrication processes extremely limited the availability and structural flexibility of the 3D microelectrodes for a wide range of biological applications.^[^ ^29^ ^]^ 3D printing techniques have shown a great promise to directly and flexibly fabricate bioelectronics with 3D bioelectrodes derived from different materials, including the organic semiconductors and carbon‐based nanomaterials.^[^ ^17^, ^30^, ^31^ ^]^ For example, Zips et al. employed ink‐jet printing to produce polyacrylate‐passivated silver conductive circuits for signal transmission, and aerosol‐jet printing to fabricate pillar‐shaped bioelectrodes using a conductive polymer ink composited of multiwalled carbon nanotubes and PEDOT: PSS with a diameter of 10 ± 2 µm and a height of 33 ± 4 µm. As a proof‐of‐concept demonstration, the multi‐material printed bioelectronics successfully record the extra‐cellular signals from a confluent layer of HL‐1 cells.^[^ ^32^ ^]^ Notably, 3D printing techniques have demonstrated to form tiny 3D bioelectronics with structural flexibility.^[^ ^33^, ^34^ ^]^ For example, Dadras‐Touss et al. demonstrated the multiphoton polymerization‐based 3D printing of bioelectronics using the PEDOT: PSS‐doped photosensitive resin. The printed 3D bioelectronics showed flexible forms, including a micro‐grid, micro‐snowflake, micro‐spring, micro‐honeycomb, and vertical micro‐tube array. Besides, they demonstrated the fabrication of a functional neural probe enabling the glucose biosensing equipped with 3D bioelectrodes shown a structural height of 7 µm and adjustable diameters from 1 to 80 µm.^[^ ^35^ ^]^ However, the existing 3D printing techniques fail to fabricate sub‐microscale or nanoscale 3D sensing bioelectrodes like conventional microfabrication technique, which limits their application for sensitively recording high‐quality intra‐cellular electrophysiological signals.^[^ ^5^, ^36^, ^37^ ^]^

Here, we present a novel multi‐material EHDP process for the production of bioelectronics with the 3D bioelectrode featuring sub‐microscale gold pillars and polymer‐passivated circuits, enabling the recording of extra‐ and intra‐cellular electrophysiological signals from electroactive cells in vitro. EHDP is employed to fabricate silver conductive circuits with microscale resolution for signal transmission, which are passivated by a thin layer of polyimide via extrusion‐based printing, and laser‐assisted EHDP is developed to produce the bioelectrode containing arrayed 3D gold pillars with sub‐microscale resolution. The printed bioelectrodes with 3D gold pillars show a high charge capacity, low impedance, and stable conductivity under the cell‐culture environment. Living cells can conform to the printed sub‐microscale 3D gold pillars to form a tight cell‐electrode interface. This feature facilitates the highly sensitive recording of weak extra‐cellular signals from cardiomyocytes and neurons. Further, by applying electroporation currents to the printed 3D bioelectrodes, intra‐cellular signals were recorded without damaging the cell viability. Moreover, the intra‐cellular recording by the printed 3D bioelectrodes was demonstrated to measure the subtle change of cellular electrophysiological activities. As a proof‐of‐concept demonstration, a fully‐printed culturing platform with four culturing chambers and tiny bioelectronics was fabricated. These individual chambers enabled chamber‐specific cell culturing and electrophysiological recording for dose‐dependent drug testing.

## 2 Results and Discussion

### 2.1 Fabrication and Characterization of the Multi‐Material EHD Printed Bioelectronics with Sub‐Microscale 3D Gold Pillars

**Figure 1:**
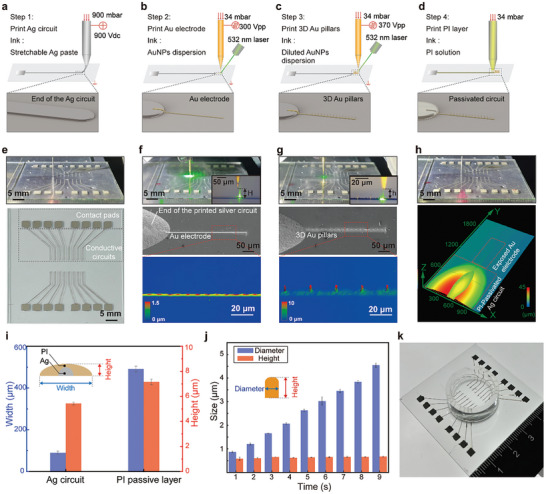
Multi‐material printing processes and structural characterization of the bioelectronics. a) Schematic of the first printing step: silver paste was printed by EHDP to form silver conductive circuits and contact pads. b) Schematic of the second printing step: the thick dispersion of gold nanoparticles was printed by laser‐assisted EHDP to form gold bioelectrodes. c) Schematic of the third printing step: the diluted dispersion of gold nanoparticles was printed by laser‐assisted EHDP to form an array of sub – microscale 3D gold pillars along the gold bioelectrode. d) Schematic of the fourth printing step: the polyimide solution was printed by extrusion‐based printing to form an insulation layer covering the silver conductive circuit. e) Photograph of the printing scene of the silver pattern and the printed silver pattern with contact pads and circuits. f) Photograph of the printing scene of the gold bioelectrode using a 5 µm inner‐diameter glass capillary, SEM image of the printed gold bioelectrode sintered with the silver conductive circuit at its end, and the inset laser confocal scanning image shows the gold bioelectrode's morphology. g) Photograph of the printing scene of the 3D gold pillars using a 1 µm inner‐diameter glass capillary, SEM image of the printed gold pillars along the gold bioelectrode, and the inset laser confocal scanning image shows the morphology of the 3D‐pillars‐decorated gold bioelectrode. h) Photograph of the printing scene of the polyimide solution, and laser confocal scanning image of the polyimide‐covered silver conductive circuit with the exposed gold bioelectrode. i) Quantification of the width and height of the printed silver conductive circuit and polyimide layer in cross‐section (n ≥ 3). j) Quantification of the diameter and height of the printed sub‐microscale 3D gold pillars (n = 3). k) Photograph of the printed bioelectronics glued with a glass ring for medium containment.

The typical form of 3D bioelectronics that enables the recording of cellular electrophysiological signals is microelectrode arrays characterized by individually addressable microelectrode sites which are composed of passivated conductive circuits and exposed 3D bioelectrodes.^[^ ^38^, ^39^ ^]^ To fabricate the 3D bioelectronics, a novel multi‐material EHDP strategy that enables the printing of the conductive silver paste, dispersions of biocompatible gold nanoparticles, and polyimide solution on a glass substrate was proposed. EHDP is based on the electrohydrodynamically induced flow of materials, enables the production of micro/nanoscale fibers in the stable cone‐jet mode or droplets in the dripping mode onto various substrates and has recently attracted extensive interest to fabricate user‐specific patterns in a controlled and high‐efficiency manner.^[^ ^40^ ^]^ Overall, the silver paste was printed via EHDP in the stable cone‐jet mode to produce microscale conductive circuits for signal transmission, and the dispersion of biocompatible gold nanoparticles was printed via laser‐assisted EHDP in the dripping mode to produce the bioelectrodes with sub‐microscale 3D gold pillars, and the polyimide solution was printed via extrusion‐based printing to produce the passivation layer (**Figure** **1**a–d).

According to the proposed multi‐material printing strategy, 16‐channel silver conductive circuits were first printed, incorporating contact pads for connecting with the external signal acquisition setup (Figure 1e; Figure S1, Supporting Information). The gold bioelectrode was printed at the end of the silver conductive circuit, with a width of 5 µm (Figure 1f; Figure S2, Supporting Information). Additionally, since the focused 532 nm laser spot sintered the gold nanoparticles in real‐time during printing, the printed gold bioelectrode was well connected with the terminal of the silver conductive circuit. As a comparison, without the sintering laser spot, the printed gold bioelectrode tended to break at the interface between the glass substrate and the silver conductive circuit (Figure S3, Supporting Information). An array of sub‐microscale 3D gold pillars was printed along the gold bioelectrode with a spacing of 25 µm (Figure 1g; Figure S7, Supporting Information). The diameter of the printed gold pillars was 0.64 ± 0.04 µm, and the height of each gold pillar was controlled by the printing time with a growth rate of ≈0.5 µm s^−1^ (Figure 1j). Thereafter, using the same moving track as the silver conductive circuit, the polyimide insulation layer was printed onto the silver conductive circuit, leaving the gold bioelectrode area exposed (Figure 1h). To demonstrate the uniformity and conformality of the printed polyimide insulation layer, we carefully cut the printed silver circuit overlaid with polyimide using a sharp blade. After exposing the cross‐section, we then observed it meticulously with SEM, and the results showed that the polyimide passivation layer was adhered uniformly to the underlying silver circuit (Figure S9, Supporting Information). Further, the size of the printed silver and polyimide tracks were characterized, the results shown that the width and height of the printed polyimide layer (width of 491.02 ± 13.26 µm, height of 7.16 ± 0.22 µm) all exceeded the silver conductive circuit (width of 89.38 ± 8.24 µm, height of 5.42 ± 0.11 µm) (Figure 1i). These results demonstrated that the underneath silver can be uniformly and completely covered by the polyimide, which can effectively shield the silver conductive circuit from direct contact with the culture medium, thereby preventing potential cytotoxic effects during cell cultures and minimizing signal leakage effects during electrophysiological signal recording. After printing the bioelectronics, a glass ring with an inner diameter of 2 cm was glued onto the substrate using polymethylsilsesquioxane (PDMS) elastomer to contain the medium (Figure 1k).

**Figure 2:**
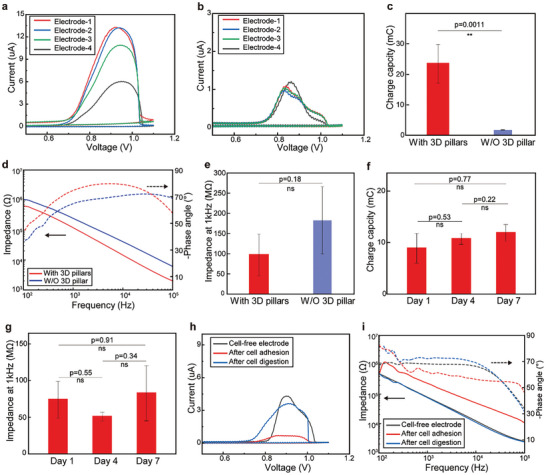
Electrochemical characterization of multi‐material printed bioelectronics. a) CV of 4 independent bioelectrodes with 3D gold pillars. b) CV of 4 independent bioelectrodes without 3D gold pillars. c) Average charge capacity of the bioelectrodes with/without 3D gold pillars (data: mean ± SD, *n* = 4, **p < 0.01). d) Impedance and phase angle spectrum (10^2^–10^5^ Hz) of representative bioelectrodes with/without 3D gold pillars. e) Average impedance of bioelectrodes with/without 3D gold pillars (data: mean ± SD, *n* = 4). f) Average charge capacity of bioelectrodes with 3D gold pillars after incubation in PBS at 37 °C for 1, 4, and 7 days (data: mean ± SD, *n* = 4). g) Average impedance at 1 kHz of bioelectrodes with 3D gold pillars after incubation in PBS at 37 °C for 1, 4, and 7 days (data: mean ± SD, *n* = 4). h) CV of the bioelectrode with 3D gold pillars before cell culture, and comparison with that of bioelectrode covered with a confluent layer of HL‐1 cardiac cells and after cell digestion. i) Impedance and phase angle spectrum (10^2^–10^5^ Hz) of bioelectrodes with 3D gold pillars before cell culture, and comparison with that of bioelectrode covered with a confluent layer of HL‐1 cardiac cells and after cell digestion.

Next, we measured and characterized the electrochemical properties of the printed bioelectronics. The cyclic voltammetry (CV) and electrochemical impedance spectroscopy (EIS) measurements were performed on the bioelectrodes with and without the sub‐microscale 3D gold pillars. The charge storage capacity of the electrode has a proportional relationship with the surface area of the CV curves, and it represents the amount of the charge transferred on the printed bioelectrode during a current‐voltage cycle.^[^ ^35^, ^41^ ^]^ As shown in **Figure** **2**a,b, the surface area of the CV curve measured from 4 independent bioelectrodes with the sub‐microscale 3D gold pillars was obviously larger than that from the bioelectrode without the sub‐microscale 3D gold pillars. According to the CV curves, the charge storage capacity of the printed bioelectrodes with/without the sub‐microscale 3D gold pillars was calculated (Equation S1, Supporting Information). The result showed that the printed bioelectrode with the sub‐microscale 3D gold pillars (23.49 ± 6.40 mC) was significantly higher than that of the bioelectrode without 3D gold pillars (1.73 ± 0.07 mC), indicating the sub‐microscale 3D gold pillars have promoted the electron transfer process on of the printed bioelectrodes (**p < 0.01) (Figure 2c). As shown in Figure 2d, the trends of both impedance magnitude and phase angle of the bioelectrode with/without 3D gold pillars were similar, indicating that both types of bioelectrodes behave like parallel capacitor‐resistor circuits, but the existence of the 3D gold pillars lowered the impedance magnitude in the frequency range of 10^2^–10^5^ Hz. Further, the average impedance magnitude of the bioelectrode with/without 3D gold pillars at 1k Hz was compared (Figure 2e; Figure S10, Supporting Information). The results shown that the bioelectrode with 3D gold pillars exhibited an impedance magnitude of 97.14 ± 51.39 MΩ at 1 kHz, which was lower than that of the bioelectrode without 3D gold pillars which showed an impedance magnitude of 182.79 ± 83.21 MΩ at 1 kHz (*p* = 0.18). To verify the stability of bioelectrodes with 3D gold pillars, the printed bioelectronics were filled with phosphate‐buffered saline (PBS) and placed in the 37 °C cell incubator, and CV/EIS were measured after being incubated for 1, 4 and 7 days (Figures S11 and S12, Supporting Information). To quantitatively illustrate the stability of the printed bioelectrode, the average charge capacity was calculated as 8.88 ± 2.89 mC, 10.72 ± 1.07 mC, and 11.86 ± 1.65 mC after being incubated for 1, 4, and 7 days, respectively, as shown in Figure 2f. Besides, the impedance magnitude at 1 kHz of the incubated bioelectrodes was 73.91 ± 25.17 MΩ, 50.74 ± 6.10 MΩ, and 82.63 ± 37.77 MΩ after being incubated for 1, 4, and 7 days, respectively, as shown in Figure 2g. The insignificantly changed charge capacity and impedance magnitude at 1 kHz for over a week demonstrated that the printed bioelectronics enables electrophysiological studies in physiological environments.

Furthermore, the CV/EIS of the cell‐free bioelectronics was compared with those of the bioelectronics cultured with a confluent layer of HL‐1 cells and after the cells were removed by trypsin (Figure S13, Supporting Information). The CV curves showed similar surface area of the cell‐free bioelectrode and the bioelectrode after cell digestion, and the average charge capacity before/after cell culture was 5.83 ± 0.48 mC and 6.78 ± 0.64 mC, showing no significant difference (Figure 2h; Figure S14, Supporting Information). The impedance and phase angle spectrums of the cell‐free bioelectrode and the bioelectrode after cell digestion were nearly overlapping, as shown in Figure 2i. These results suggest that the printed bioelectronics are stable after cell culture and can be reused after remove the adhered cells. Additionally, the bioelectrode after cell adhesion showed CV curve with largely decreased surface area compared to the other groups, and exhibited a significantly lower charge capacity of 1.90 ± 0.37 mC (Figure S14, Supporting Information). Besides, the impedance spectrum of the bioelectrode after cell adhesion was shifted to higher impedance values, and the trend of its phase angle spectrum was also different from the compared to the other two groups (Figure 2i). These difference in CV/EIS plots demonstrated cell adherence onto the printed bioelectronics, and proved the possibility of the printed bioelectronics to study cell adherent status.^[^ ^42^ ^]^

### 2.2 Characterization of the Cell‐Electrode Contact Interface and the Extracellular Recording of the Multi‐Material EHD Printed Bioelectronics with Sub‐Microscale 3D Gold Pillars

**Figure 3:**
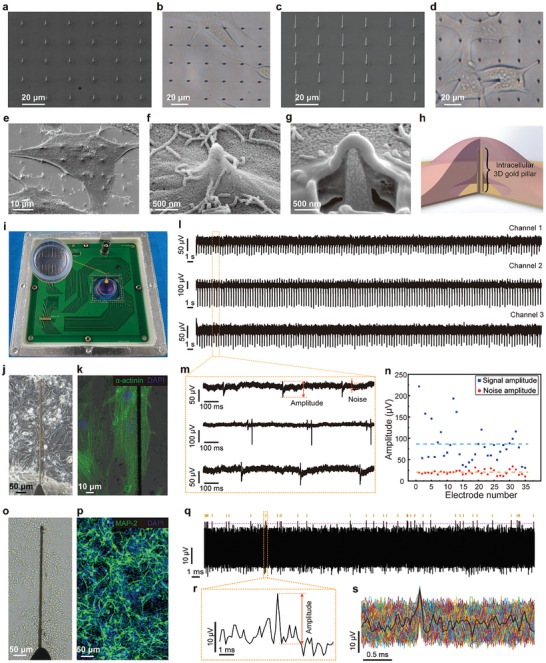
Characterization of the interfacing relationship between the printed 3D sub‐microscale gold pillar and cell and electrophysiological recording using the printed bioelectronics. a) SEM image shows the printed array of short gold pillars with a height of 5 µm on a glass substrate, and b) an optical image of the HL‐1 cells growing atop the 3D gold pillars. c) SEM image shows the printed array of tall gold pillars with a height of 10 µm on a glass substrate, and d) an optical image of HL‐1 cells growing on the planar substrate among the pillars. e) SEM image shows a tilted view of an HL‐1 cell attaching to an array of short gold pillars. f) Enlarged SEM image showing the local cell membrane wrapping on the gold pillar. g) FIB‐SEM image showing the interface between the local cell membrane and the underlying gold pillar. h) Schematic illustrates the relationship between the cell and the printed 3D gold pillar. i) The printed bioelectronics assembled in a customized PCB. j) Optical image showing printed bioelectrode covered by a confluent layer of primary cardiomyocytes. k) Fluorescent image showing the functional cardiomyocyte growing on a bioelectrode, staining with α‐actinin and DAPI. l) Representative extracellular electrophysiological signals recorded by three individual bioelectrodes, and m) enlarged waveform of these signals. n) Distribution of signal and noise amplitudes, each dot indicates the values observed on an individual bioelectrode, and the mean values are indicated by the dashed lines. o) Optical image showing printed bioelectrodes covered by a confluent layer of neurons. p) Neuronal subtype analysis, staining with MAP‐2 and DAPI. q) Spontaneous neuronal activity recorded from the bioelectrode, showing detected spikes and corresponding raster plot (the red dash indicating the selected threshold). r) A segment from the recorded signal showing an individual spike. (s) Overlay of all detected spikes with a mean waveform in black.

To assess the impact of the printed sub‐microscale 3D gold pillars on cell growth, we cultured HL‐1 cells onto glass substrates printed with sub‐microscale 3D gold pillars with a height of ≈5 and 10 µm (**Figure** **3**a,c). After achieving cell adhesion through a 6‐h culture, the overall cell morphology was evaluated using optical imaging. The results showed that cells cultured on the substrate printed with sub‐microscale 3D gold pillar arrays with a height of 5 µm exhibited a fully spread morphology but the spreading of cells cultured on the substrate printed with sub‐microscale 3D gold pillar arrays with a height of 10 µm was hindered by pillars (Figure 3b,d). Further, to investigate the interface between the cell and the printed sub‐microscale 3D gold pillars, samples were dehydrated and observed with SEM. The SEM view demonstrated that the cell was grown atop the printed sub‐microscale 3D gold pillars with a height of 5 µm, and the enlarged view illustrated that the cell membrane bent locally at the pillar position (Figure 3e,f). As a comparison, the cell grew on the substrate printed with sub‐microscale 3D gold pillars featuring a height of 10 µm was adhered onto the planar substrate rather than covering onto the pillars (Figure S15, Supporting Information). To further investigate the underlying interface between the local cell membrane and the printed 3D gold pillar, focused ion beam (FIB) milling was applied to expose the underlying interface between the cell and pillar. Subsequent SEM imaging demonstrated that the local membrane of HL‐1 cell formed a tight interface with the underlying 3D gold pillar (Figure 3g,h). Additionally, to reveal the interface between the printed 3D gold pillars and living cells, HL‐1 cells cultured on an array of 3D gold pillars with a height of 5 µm were stained with vital cell membrane dye, and the results showed that bright red dots on the membrane of a living HL‐1 cell appeared at the location of 3D gold pillars, indicating the adherence of cells atop the printed pillars (Figure S16, Supporting Information).^[^ ^43^ ^]^ Consequently, the printed sub‐microscale 3D gold pillar with a height below 5 µm can be applied for further living‐cell electrophysiological signal recording experiments.

To evaluate the extracellular electrophysiological recording function, primary cardiomyocytes, and neurons that can spontaneously generate electrophysiological signals were cultured on the printed bioelectronics with sub‐microscale 3D gold pillars. Primary cardiomyocytes plated on the printed bioelectronics reached a confluent layer after being cultured for 48 h (Figure 3j). The fluorescent image reflected that the cardiomyocyte growing on the printed bioelectrode with 3D gold pillars exhibited fully spread morphology (Figure 3k). The cell‐laden bioelectronics with 16 independently addressable channels was assembled with a customized printed circuit board (PCB) to carry out electrophysiological signal recording, and a 4 by 4 map shows the most channels of the printed bioelectronics were able to record the extracellular electrophysiological signals (Figure 3i; Figure S17, Supporting Information). Representative signals demonstrated that the amplitude of spikes generated by cardiomyocytes is much lower than the baseline noise (Figure 3l,m). To further illustrate this, the amplitude of the spike and noise of signals recorded by 35 independently addressable channels from four printed bioelectronics was compared, and the amplitude of the spike (84.57 ± 44.72 µV) was significantly higher than the noise (20.04 ± 5.73 µV) (Figure 3n). These results indicated extracellular signals of cardiomyocytes can be effectively recorded by the printed bioelectronics.

Moreover, neurons that generate extracellular electrophysiological signals with lower amplitude were cultured on the printed bioelectronics.^[^ ^44^, ^45^ ^]^ After a 6‐day plating and 8‐day differentiation, a confluent layer of neurons was developed on the bioelectronics, characterized by the expression of MAP‐2 biomarker (Figure 3o,p). Signals recording revealed spontaneous neuronal activity, the recorded neuronal spike exhibited a low spike amplitude of ≈15 µV (Figure 3q,r). Besides, the spikes recorded by a printed bioelectrode during a single recording session were aligned, and the reproducible signal spikes exhibited the similar pattern as neural spikes in reported works, which demonstrated the spikes recorded by the printed bioelectronics were in fact neural spikes (Figure 3s).^[^ ^44^, ^46^ ^]^ These results further demonstrated the feasibility of the printed bioelectronics for recording the extracellular electrophysiological signals generated by electroactive cells.

### 2.3 Intracellular Electrophysiological Recording of the Multi‐Material EHD Printed Bioelectronics with Sub‐Microscale 3D‐Printed Pillars

**Figure 4:**
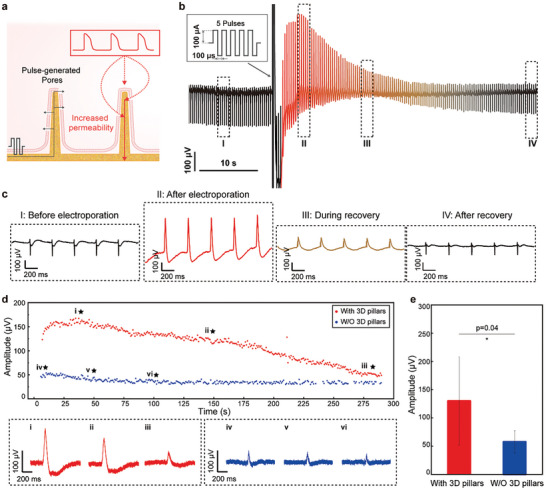
Intra‐cellular signal recording using bioelectronics with sub‐microscale 3D gold pillars. a) Schematic illustrating the increased cell membrane permeability induced by electroporation at the 3D pillars. b) Transient intra‐cellular recording of cardiomyocytes after electroporation. c) Enlarged view of extra‐cellular and intra‐cellular signal forms from the cardiomyocyte before and after electroporation. d) The amplitude evolution of the intracellular electrophysiological signals recorded from primary rat cardiomyocytes using the gold bioelectrode with and without 3D gold pillars. The typical spikes recorded by the gold bioelectrode with and without 3D gold pillars occurring at the star‐marked time are indicated in insets (i–iii) and (iv–vi). e) Comparison of the peak amplitudes of intracellular electrophysiological signals recorded by the gold bioelectrode with and without 3D gold pillars (data: mean ± SD, *n* = 7, *p* = 0.04).

As previously discussed, the measurement of extracellular signals was realized using printed bioelectronics with sub‐microscale 3D gold pillars. Further, transient electroporation current pulses were sent to the bioelectrode with sub‐microscale 3D gold pillars to create tiny pores on the cell membrane and establish an intracellular access (**Figure** **4**a).^[^ ^47^ ^]^ A biphasic 5‐pulse current train with an amplitude of 100 µA was selected as the proper electroporation current for the printed bioelectronics (Figure S18, Supporting Information). After sending the electroporation current to the bioelectrode with sub‐microscale 3D gold pillars, the recorded signal wave switched instantly from the extracellular to intracellular signal form, with the intracellular signal showing a significantly higher amplitude than the extracellular one (Figure 4b). The amplitude of the recorded intracellular signal gradually declined with time and the signal shape was observed to transit back to the extra‐cellular signal form (Figure 4c). The recovery of the recorded signal was caused by the self‐resealing of pores generated on the cell membrane, and this phenomenon can evidence that the intracellular recording using the printed bioelectronics with 3D gold pillars does not damage the activity of cardiomyocytes.^[^ ^11^ ^]^ Due to the non‐damage process, the intracellular recording induced by the printed bioelectronics with 3D gold pillars proved to be repeatable (Figures S19 and S20, Supporting Information). In the example, the extra‐cellular signals showed amplitude of 85.31 ± 9.82 µV, and intra‐cellular signals with peak amplitude of 246.79 ± 74.62 µV were recorded right after the first electroporation event. As the pores on the cellular membrane were resealed, the signal amplitude decreased, and the shape of the extra‐cellular signal started to be noticeable. At this point, the current pulse train was sent to the bioelectrode again, and intracellular signals with peak amplitude of 331.38 ± 21.64 µV were recorded.

To illustrate the function of the sub‐microscale 3D gold pillars in enhancing the signal quality, we compared the quality of signals recorded by the gold bioelectrode with and without 3D pillars. In the presented results, the signal amplitude recorded by the gold bioelectrode with 3D pillars was continuously higher than that recorded by the gold bioelectrode without 3D pillars (Figure 4d). In addition, the peak amplitude of the intracellular signal recorded by the gold bioelectrode with 3D pillars in Figure 4d(i) exceeded 150 µV, which was approximately three times as high as that recorded by the gold electrode without 3D pillars in Figure 4d(iv). Moreover, the average peak amplitude of the intracellular signal recorded by the gold bioelectrode with 3D pillars was significantly higher than that without 3D pillars, being 130.12 ± 77.86 µV and 57.80 ± 19.47 µV (*p* = 0.04, Figure 4e). These results indicate an improved intracellular signal quality due to the existence of the printed 3D pillars.

**Figure 5:**
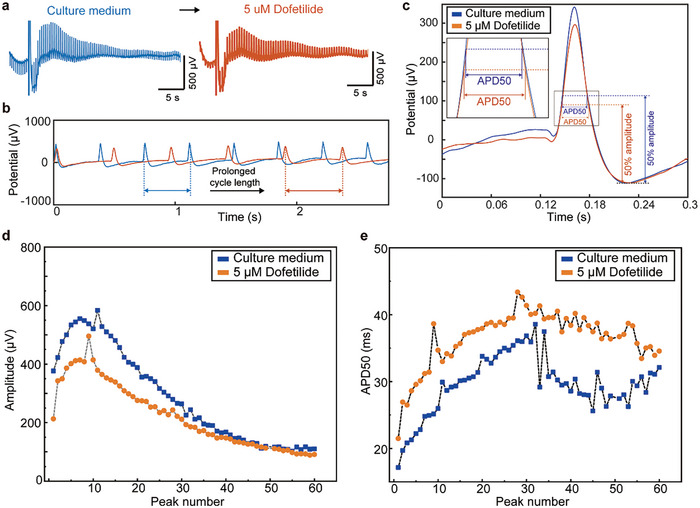
Revealing the effects of the ion channel blocker using the intra‐cellular signals recorded by the bioelectronics with sub‐microscale 3D gold pillars. a) Intra‐cellular signals before and after the treatment of dofetilide, and b) an enlarged view of these signals. c) Overlaid view showing a pair of individual intra‐cellular signal spikes before and after dofetilide treatment. Quantitative comparison between the d) amplitude and e) APD 50 value before and after dofetilide treatment.

The recorded intra‐cellular signals using printed bioelectronics with sub‐microscale 3D gold pillars exhibited the feature of real action potentials of cardiomyocytes, which can reveal detailed information about cellular electrophysiological activities.^[^ ^48^, ^49^ ^]^ To demonstrate the function of the recorded intra‐cellular signals, cardiomyocytes cultured in the printed bioelectronics with sub‐microscale 3D gold pillars were treated with 5 µm dofetilide, which is a known potassium ion channel blocker that reduces potassium conductance during repolarization and lengthens the action potential duration (APD)^[^ ^47^, ^50^ ^]^ (**Figure** **5**a). By enlarging and aligning the recorded intra‐cellular signal with and without the dofetilide treatment, it is clearly shown that the recorded intra‐cellular signals exhibited prolonged cycle length after the drug treatment (Figure 5b). Further, to demonstrate the function of analyzing the shape of the recorded intra‐cellular signal, signal amplitudes and APD50 values (a parameter recognized as the time interval where the action potential is more negative than 50% repolarization^[^ ^5^, ^51^ ^]^) of the recorded intra‐cellular signal with and without the dofetilide treatment were compared. The results indicated that the dofetilide treatment induced a reduction of the peak amplitude and a prolonged APD50 value (Figure 5c). The quantitative calculation revealed that the amplitude decreased by ≈18% while the APD50 value increased by ≈24% (Figure 5d,e), which is consistent with previous research.^[^ ^11^ ^]^ These results demonstrated the possibility of printed bioelectronics with sub‐microscale 3D gold pillars to measure the subtle change of cellular electrophysiological signals.

### 2.4 Fully 3D‐Printed Chips with Culturing Chambers and Bioelectronics

**Figure 6:**
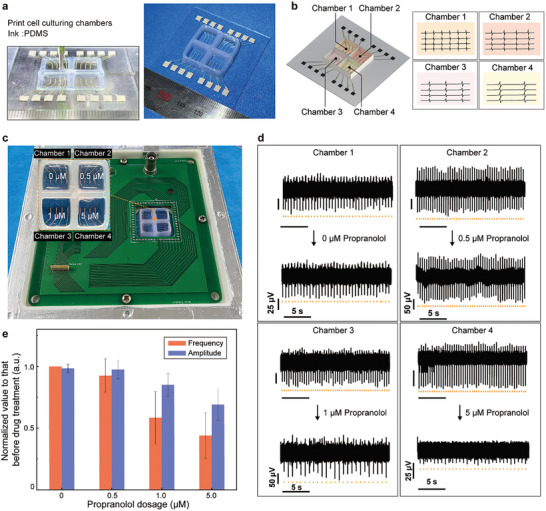
Printing of cell culturing chambers and validation of the sectionalized electrophysiological signal recording function of the multi‐material‐printed bioelectronic chip. a) Extrusion‐based printing of the PDMS ink, forming four cell culturing chambers. b) Schematic illustrating the sectionalized recording function benefiting from the four independent culturing chambers. c) The photograph shows a fully‐printed chip mounted in the PCB, and the inset image illustrates different drug‐dose treatments in individual culturing chambers. d) Sectionalized drug treatment and independent electrophysiological recording on the same chip. The triangular orange marks are corresponding to the spikes of the signal. e) Quantitative comparison among the firing frequency and signal amplitude of the recorded signal under different drug treatments (data: mean ± SD, *n* = 3).

The printed bioelectronics applied a glass‐ring chamber as the cell culturing chamber. However, the glass‐ring chamber was glued onto the substrate separately, and the single‐chamber chip is not convenient for the drug test that commonly involves multiple dosages.^[^ ^50^ ^]^ To produce fully‐printed chips with culturing chambers and bioelectronics, a printing step for producing culturing chambers was developed following the four printing steps for producing the bioelectronics. Four individual culturing chambers were printed using the PDMS ink (**Figure** **6**a). Additionally, culturing chambers made of biocompatible thermoplastic polymers (such as polycaprolactone, as shown in Figure S21, Supporting Information) were also printed as they are less prone to bulk absorption of hydrophobic drugs than PDMS.^[^ ^52^ ^]^ The four individual PDMS culturing chambers divided the 16 electrodes into four sections, enabling the electrophysiological recording from each section independently (Figure 6b).

To validate the sectionalized recording function of the printed bioelectronics, primary cardiomyocytes were plated in the four individual culturing chambers for 48 h, and the printed bioelectronic device was mounted on the customized PCB. Thereafter, different doses of propranolol, a known cardiac‐targeted drug inhibiting the activation of cardiomyocytes, were added to these separated culturing chambers (Figure 6c). Figure 6d shows representative electrophysiological signals recorded during the dose‐dependent test, and the results indicated that the propranolol‐induced reduction in the firing frequency and signal amplitude was in correlation with the drug dose. The chamber‐1 (0 µm) as the control group showed no significant change in the signals. From chamber‐2 (0.5 µm) to chamber‐4 (5 µm), the higher dose induced a more significant reduction in the frequency and amplitude. Further, quantitative analysis of the dose‐dependent test indicated that in response to 0.5, 1, and 5 µm propranolol, the firing frequency changed to ≈92.6 ± 13.4%, 58.5 ± 18.4%, and 43.7 ± 18.3%, and the signal amplitude changed to ≈97.5 ± 7.7%, 85 ± 9.2%, and 69 ± 12.6% of that before the dose treatment, respectively (Figure 6e; Table S1, Supporting Information). These results demonstrated that the printed bioelectronics enabled independent electrophysiological signal monitoring from the four culturing chambers without affecting each other, which is useful in drug‐dose tests.

## 3 Conclusion

In this work, a novel multi‐material EHDP strategy is proposed to produce polymer‐passivated bioelectronics with sub‐microscale 3D gold pillars for in vitro electrophysiological recording. EHDP is employed to fabricate microscale conductive circuits for signal transmission, which is passivated by a thin layer of polyimide via extrusion‐based printing. Laser‐assisted EHDP is developed to produce the bioelectrodes containing arrayed 3D gold pillars with a diameter of 0.64 ± 0.04 µm. Additionally, the EHDP process enables the customization of the printed 3D sub‐microscale structures, which can facilitate the research of the dynamic interface between the cellular membrane and guide the design of novel bioelectronics. The printed bioelectrodes with 3D gold pillars show a high charge capacity, low impedance, and stable conductivity under the cell‐culture environment. Living cells conformally adhere onto the sub‐microscale 3D pillars with a height below 5 µm, forming a tight cell‐electrode contact interface. This printed bioelectronics enables the recording of extra‐cellular electrophysiological signals. Further, the 3D pillars can apply electroporation currents to reversibly open the cellular membrane for intra‐cellular signal recording, enabling to measure the subtle change of electrophysiological signals. As a proof‐of‐concept demonstration, a fully‐printed culturing chip with multiple culturing chambers and tiny bioelectronics is fabricated to enable chamber‐specific cell culturing and electrophysiological recording for dose‐dependent drug testing. The proposed multi‐material EHDP provides an innovative strategy for the rapid prototype of organ‐on‐a‐chip systems with multiscale bioelectronics for non‐destructive in situ recording of extra‐ and intra‐cellular electrophysiological signals.

## 4 Experimental Section

### Materials and Equipment Setup for the Multi‐Material Printing Process

The stretchable silver paste (S201, Beijing NanoTop Electronic Technology Co., Ltd., China) was applied for printing silver conductive circuits. The concentrated dispersion of gold nanoparticles showing an average diameter of 3.4 ± 0.96 nm with 50 wt% (Sigma, lot. 122 409) was applied to print gold bioelectrodes. Diluted dispersion of gold nanoparticles was prepared by mixing the concentrated dispersion with N‐tetradecane in a mass ratio of 1:7 and stirred overnight before printing the sub‐microscale 3D gold pillar. Dilution was necessary to avoid clogging in the sub‐microscale 3D gold pillar printing process.^[^ ^53^ ^]^ The polyimide ink (Dongguan Donglin Polymer Materials Co., Ltd., Shaanxi, China) was used to print the insulation layer. The PDMS ink (SE1700, Dow‐Corning) was mixed with a curing agent with a mass ratio of 1:10 for printing cell culturing chambers.

The multi‐material printing process was performed using an EHD printer (Shaanxi Baipusheng Medical Technology Development Co., Ltd., Shaanxi, China) equipped with a high‐resolution XYZ moving module, a high‐voltage module including a high‐voltage supplier (Dong Wen High Voltage, Tianjin, China) and a signal generator (RIGOL), a gas pressure controlling module including a pump and a pneumatic value (Fluent, France), and two cameras were set along the X and Y axis for real‐time monitoring of the printing process. The printing parameters and patterns were controlled by built‐in software.

### EHDP of the Silver Conductive Circuits

To print silver conductive circuits, a 25 G stainless nozzle was mounted in the printer. After loading with the stretchable silver ink, the nozzle was connected to the voltage supplier and gas supplier. To stably extrude the silver paste from the nozzle, the gas pressure was set of 900 mbar. Further, the nozzle‐to‐substrate distance was set of 300 µm and a high direct current (DC) voltage was set of 900 V to induce the formation of a Taylor cone jet. The pattern of silver conductive circuits was determined by the movement of the moving module according to user‐defined programs.

### Laser‐Assisted EHDP of the Bioelectrode and the Sub‐Microscale 3D Gold Pillar

The 3D bioelectrode consists of the bottom gold bioelectrode and on‐top sub‐microscale 3D gold pillars. Glass capillaries with inner diameters of ≈5 and 1 µm were prepared using a micropipette pulling system (Sutter Instrument) to print the gold bioelectrode and 3D pillars (Figure S4, Supporting Information). Prior to printing, the outer surface of the glass capillary was plated with chrome (with a thickness of 10 nm) and gold (with a thickness of 100 nm) by magnetron sputtering to obtain electrical conductivity.

To print the bottom gold bioelectrode, the thick dispersion of gold nanoparticles was loaded in the glass capillary with an inner nozzle diameter of 5 µm, and the capillary was connected to the voltage supplier and gas supplier. A gas pressure of 34 mbar was set to deliver the ink to the nozzle tip, and the distance between the nozzle tip and the substrate was ≈50 µm. Further, a monophasic pulse voltage (with an amplitude of 300 V and frequency of 10 kHz) was set to drive the ink drops from the nozzle tip to the glass substrate. The 532 nm green laser with the power of 4–6 mW was focused on the landing point of the ink drops to sinter the gold nanoparticles in real‐time. To make the gold electrode structure in the form of a continuous line, the reciprocating movement of 200 times was used.

To print the sub‐microscale 3D gold pillars, the diluted dispersion of gold nanoparticles was loaded in the glass capillary with an inner nozzle diameter of 1 µm (Figure S4, Supporting Information). A gas pressure of 34 mbar was set to drive the ink to the tip of the nozzle, and the distance between the nozzle tip and the substrate was ≈30 µm. Further, a monophasic pulse voltage (with an amplitude of 370 V and frequency of 1 kHz) between the nozzle tip and the substrate was set to drive the ink drops from the nozzle tip to the landing site, and a 532 nm green laser spot with diameter <10 µm was focused on the landing point of the ink drops to sinter the gold nanoparticles in real‐time (Figure S5, Supporting Information).

To print the sub‐microscale 3D gold pillars on the bottom gold bioelectrode, the printing nozzle and the laser spot were staying directly above the printed bottom gold bioelectrode for a certain time, and an up‐grown pillar with a certain height was generated at the printing location. After one 3D gold pillar was finished, the printing nozzle and the laser spot were moved to the next printing position pre‐designed along the bottom gold bioelectrode (Figure S7, Supporting Information).

### Extrusion‐Based Printing of the Passivation Polyimide Layer and Cell Culturing Chambers

The polyimide passivation layer was printed onto the silver conductive circuit, leaving the gold bioelectrode at the end of the circuits exposed. To print the polyimide passivation layer, a 25 G stainless nozzle (with an inner diameter of 260 µm) was mounted in the printer. After loading with the polyimide ink, the nozzle was connected to the gas supplier. To stably extrude the polyimide ink from the nozzle, the gas pressure was fixed at 34 mbar, and the moving track of the nozzle was the same as the trajectory of the printed silver conductive circuits.

The cell culturing chambers divided the 16‐channel microelectrodes into four individual zones. For the printing of culturing chambers, a 15 G stainless nozzle (with an inner diameter of 1.43 mm) was used. After loading with the PDMS ink, the nozzle was connected with the gas supplier and 1500–1600 mbar gas pressure was applied to extrude the PDMS ink. The moving speed was adjusted to 4–5 mm s^−1^ to ensure a continuous printing process.

### Structural and Electrochemical Characterization of the Printed Bioelectronics with Sub‐Microscale 3D Gold Pillars

The width of the printed silver conductive circuit and polyimide passivation layer were measured using the inverted fluorescence microscope (Nikon Ti‐S, Japan). The morphology of the printed sub‐microscale 3D gold pillar was observed by SEM (Hitachi S‐3000N, Japan) and laser scanning confocal microscopy (OLS4000, Japan).

A commercial potentiostat (Autolab PGSTA302N, Metrohm) was used to measure the CV and impedance spectrum of the printed microelectrode arrays. Each measurement was carried out by placing the measuring electrode from the potentiostat in contact with the contact pad connecting with a printed gold bioelectrode. The cell culture chamber was filled with 5 mm K_3_[Fe(CN)_6_]/K_4_[Fe(CN)_6_] in 100 mm KCl solution and the platinum reference electrode from the potentiostat was submerged in the solution. The CV curves were recorded by cycling the potential of the working electrode with a scan rate of 100 mV s^−1^. Impedance spectroscopy was performed by applying a sine signal with frequencies ranging from 10^2^ Hz to 10^5^ Hz.

To test the long‐term stability under cell‐culture conditions, impedance spectroscopy was carried out by immersing the chips in PBS at 37 °C and 5% CO_2_ in a humidified incubator for up to 7 days. The data was recorded with a controller board (RHS2000, Intan Technologies, Los Angeles, USA) and amplifier chips (RHS2116, Intan Technologies, Los Angeles, USA).

### Culture and Characterization of Primary Cardiomyocytes and HL‐1 Cells

Primary cardiomyocytes were isolated from the Sprague‐Dawley (SD) rat with the age of 1–2 days as previously described.^[^ ^54^ ^]^ All animal experiment procedures were conducted according to the National Institutes of Health Guidelines for Care and were accepted by the Use of Laboratory Animal Care and Use Committee of Xi'an Jiaotong University (2021‐1140, Xi'an, China). Primary cardiomyocytes were plated on the multi‐material printed bioelectronics with a cell density of 3000 cells/mm^2^. Before plating the cardiomyocytes, the culture chamber was treated with oxygen plasma for 5 min and the substrate was functionalized with 1 mg ml^−1^ bovine fibrinogen solution (solved in Dulbecco's PBS), incubated at 37 °C for 2 h, then gently washed with PBS right before cell seeding.

To characterize the primary cardiomyocytes by immunofluorescence, samples were rinsed three times with DPBS and then fixed with 4% paraformaldehyde for 20 min at room temperature. For the immunostaining of α‐actinin, the samples were immersed with sarcomeric α‐actinin (1:200 dilution, Abcam, no. ab9465) at 4 °C overnight. Then, Alexa Flour‐488 conjugated secondary antibody (1:1000 dilution, Invitrogen, no. A‐11029) was added and incubated for 1.5 h at room temperature. Cell nuclei were stained with DAPI (Servicebio, no. G1012) for 5 min. The fluorescence images of the stained sample were viewed with an inverted laser confocal microscope (Nikon, Japan).

The HL‐1 cells (TCM‐C783, Haixing Biosciences, Suzhou, China) were seeded on glass substrates printed with sub‐microscale 3D gold pillar arrays with a density of 2500 cells/cm^2^. Prior to cell seeding, glass substrates were treated with oxygen plasma for 5 min and the substrate was functionalized with 1 mg ml^−1^ bovine fibrinogen solution.

To stain the HL‐cells with Cell Mask Deep Red dye (Thermo Fisher Scientific Inc., C10046), samples were washed with 37 °C preheated DPBS buffer for three times and then incubated with 0.25 µg ml^−1^ Cell Mask Deep Red dye for 10 min in 37 °C incubator. After removing the dye and washing the sample with the DPBS buffer for three times, the fluorescence images of the stained sample were viewed with an inverted laser confocal microscope (Nikon, Japan).

### Hardware Setup for In Situ Electrophysiological Recording

All electrophysiological signals were obtained with a controller board (RHS2000, Intan Technologies, Los Angeles, USA) and amplifier chips (RHS2116, Intan Technologies, Los Angeles, USA). A PCB was designed using the KiCad software. The PCB contained 2 connectors (A79024, Omnetics connector corporation) and 60 spring probe electrodes (POGO PIN, SZXHN, Shenzhen, China) to connect the contact pads and the amplifier chips. During electrophysiological recording, the data acquisition system was controlled by Intan stimulation/recording controller software. The data recorded from the stimulation/recording controller software were loaded into MATLAB (The Mathworks, USA) with an open‐source m‐code function provided by Intan Technologies. The data were further analyzed using Origin (OriginLab Corporation, USA) and MATLAB.

### SEM Imaging of the Printed Bioelectronics and Cells

Cells were fixed with 4% paraformaldehyde for over 30 min, and the samples can be preserved at 4 °C. The dehydration was accomplished by different incubations of ethanol for 10 min at 4 °C. The ethanol concentration was increased from 30 v/v% of ethanol diluted in water, then 50 v/v%, 70 v/v%, 90 v/v%, and twice 100 v/v% of ethanol. The sample in 100% ethanol was dried with liquid CO_2_ in a critical point drier, which preserved the cell morphology during the drying step. A thin layer of gold was sputtered on the sample using the sputter coater before SEM imaging (Hitachi S‐3000N, Japan). Samples were loaded into the vacuum chamber of the SEM, and the region of interest was selected through scanning electron beam imaging.

### Characterization of the Interface between the Cell Membrane and the Sub‐Microscale 3D Gold Pillars

The interface between the cell membrane and the sub‐microscale 3D gold pillars was characterized using FIB‐SEM. Prior to the FIB‐SEM observation, the sample was pretreated according to the SEM imaging procedure. Generally, the FIB‐SEM imaging process includes locating, electron‐beam‐induced platinum deposition, I‐beam platinum deposition, cross‐section etching, and cross‐section observation. The sample was mounted on the carrier stage and loaded into the vacuum chamber of the dual‐beam microscope (Helios Nanolab600i, Thermo Fisher Scientific Inc., Massachusetts, USA). For locating, the surface of the sample was observed under SEM imaging mode, and the area of interest was located and imaged. For electron‐beam‐induced platinum deposition, the platinum gun is placed into the chamber, platinum deposition is triggered by an electron beam, and a platinum layer with a thickness of 1 µm is coated onto the area to be observed. For I‐beam platinum deposition, the carrier stage was tilted by 52° and the position of the carrier stage was adjusted to avoid knocking against the lens and detectors, and the second platinum deposition was triggered by the I‐beam. For cross‐section etching, the etching area was selected within the platinum‐deposited region through the software, and a trench was etched by the I‐beam at the selected area to expose the cross‐section. This procedure can be repeated several times to reproduce the interface between the cell membrane and the sub‐microscale 3D gold pillars. For cross‐section observation, the etched area of the sample was observed under SEM imaging mode.

### Statistical Analysis

Experiments were repeated in triplicate for each sample, and the quantified data were presented as mean ± standard deviation. Statistical differences were obtained through analysis of variance followed by Tukey's significant difference post hoc test. A significance level of 0.05 was applied to determine significant differences.

## Conflict of Interest

The authors declare no conflict of interest.

## Supporting information



Supporting Information

## Data Availability

The data that support the findings of this study are available from the corresponding author upon reasonable request.
