# Supplementary material for: Multi‐material Electrohydrodynamic Printing of Bioelectronics with Sub‐Microscale 3D Gold Pillars for In Vitro Extra‐ and Intra‐Cellular Electrophysiological Recordings
Source: Adv Sci (Weinh). 2025 Jan 10;12(9):2407969. doi: 10.1002/advs.202407969 (PMC11884540; doi:10.1002/advs.202407969)
Supplement: Supplementary file 1 — Supporting Information [file ADVS-12-2407969-s001.docx]

# Supplementary Information

# Multi-material electrohydrodynamic printing of bioelectronics with sub-microscale 3D gold pillars for *in vitro* extra- and intra-cellular electrophysiological recordings

Bingsong Gu^1, 2, 3^, Qihang Ma^1, 2, 3^, Jiaxin Li^1, 2, 3^, Wangkai Xu^1, 2, 3^, Yuke Xie^4^, Peng Lu^4^, Kun Yu^1, 2, 3^, Ziyao Huo^1, 2, 3^, Xiao Li^1, 2, 3^, Jianhua Peng^4^, Yong Jiang^4^, Dichen Li^1, 2 ,3^, and Jiankang He^1, 2, 3, *^

^1^ State Key Laboratory for Manufacturing Systems Engineering, Xi’an Jiaotong University, Xi’an, 710049, People’s Republic of China

^2^ National Medical Products Administration (NMPA) Key Laboratory for Research and Evaluation of Additive Manufacturing Medical Devices, Xi’an Jiaotong University, Xi’an 710049, People’s Republic of China

^3^ National Innovation Platform for Industry-Education Integration of Medical Technology, Xi'an Jiaotong University, Xi’an 710049, People’s Republic of China

^4^ Laboratory of Neurological Diseases and Brain Function, The Affiliated Hospital of Southwest Medical University, Luzhou 64600, People’s Republic of China

* Corresponding author at: State Key Laboratory for Manufacturing Systems Engineering, Xi’an Jiaotong University, Xi’an 710049, People’s Republic of China

E-mail address: jiankanghe@mail.xjtu.edu.cn (J. He)


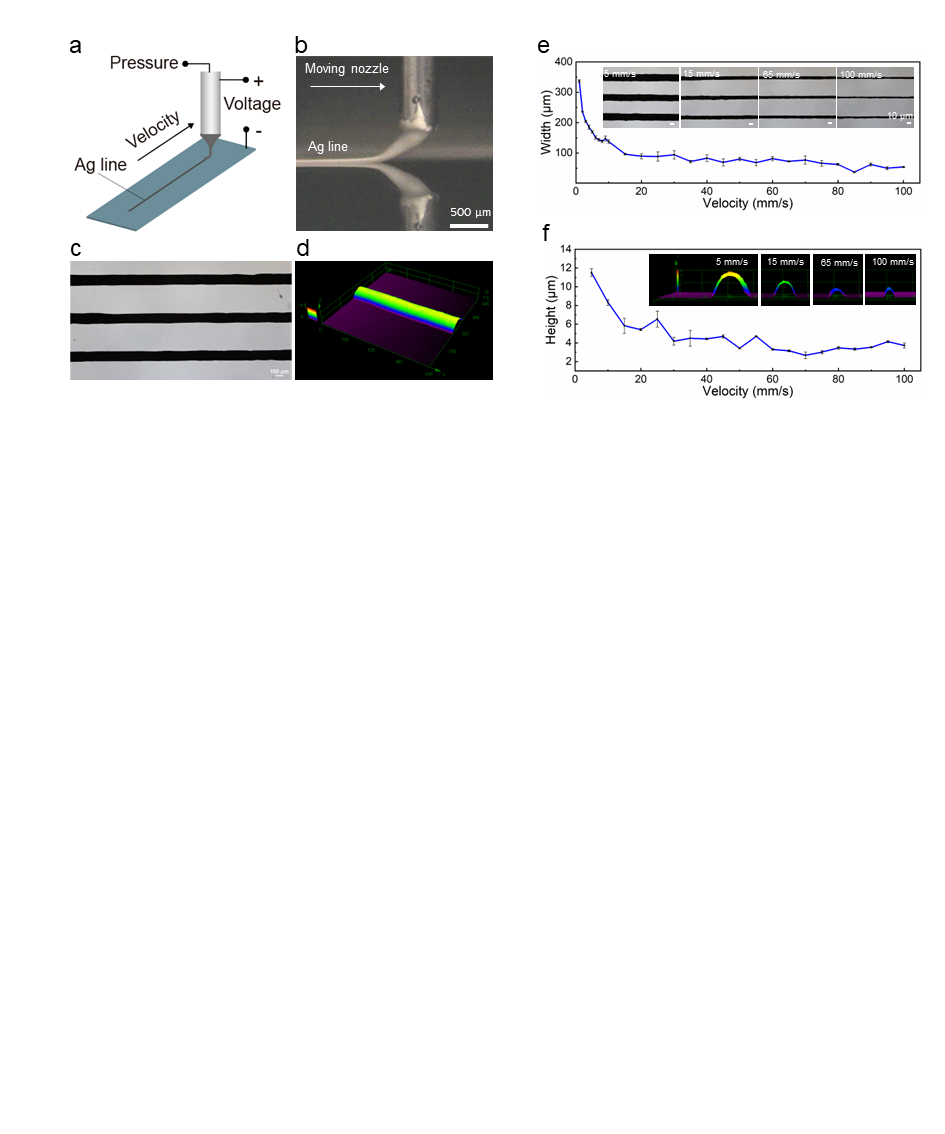


Figure S1. Electrohydrodynamic printing (EHDP) of silver lines using the stretchable silver paste. (a) Schematic and (b) photograph illustrate the EHDP process of the silver line. (c) Optical image and (d) laser confocal image showing the printed silver line. (e) The relationship between the moving velocity of the printing nozzle and the width of the printed silver lines. (f) The relationship between the moving velocity of the printing nozzle and the height of the printed silver lines.


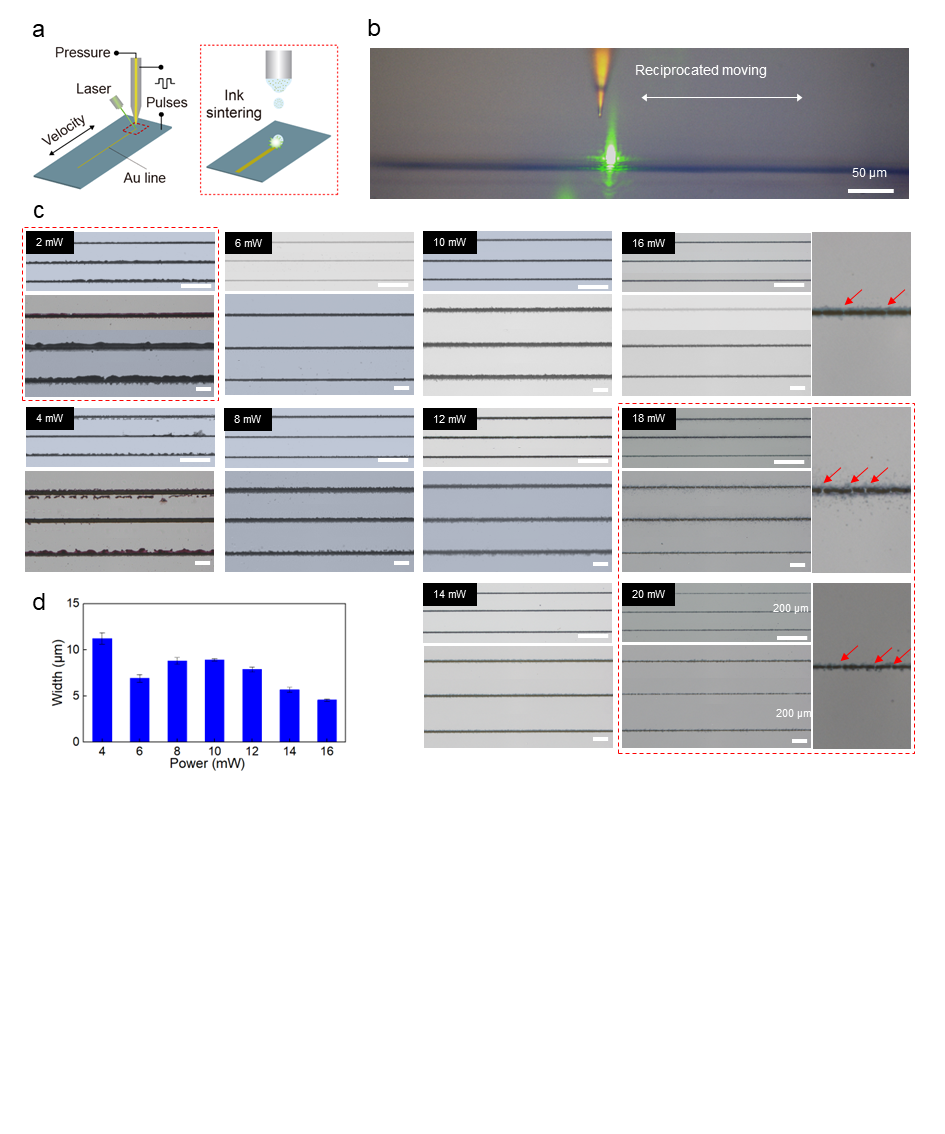


Figure S2. Laser-assisted EHDP of gold lines using the dispersion of gold nanoparticles. (a) Schematic and (b) photograph illustrates the laser-assisted EHDP process of the gold line. (c) Optical images show the printed gold lines using laser power from 2 mW to 20 mW. The gold lines printed using 2 mW show spread gold nanoparticles dispersion while the gold lines printed using 18 mW and 20 mW show breaks. (d) The relationship between the laser power and the width of the printed gold lines.


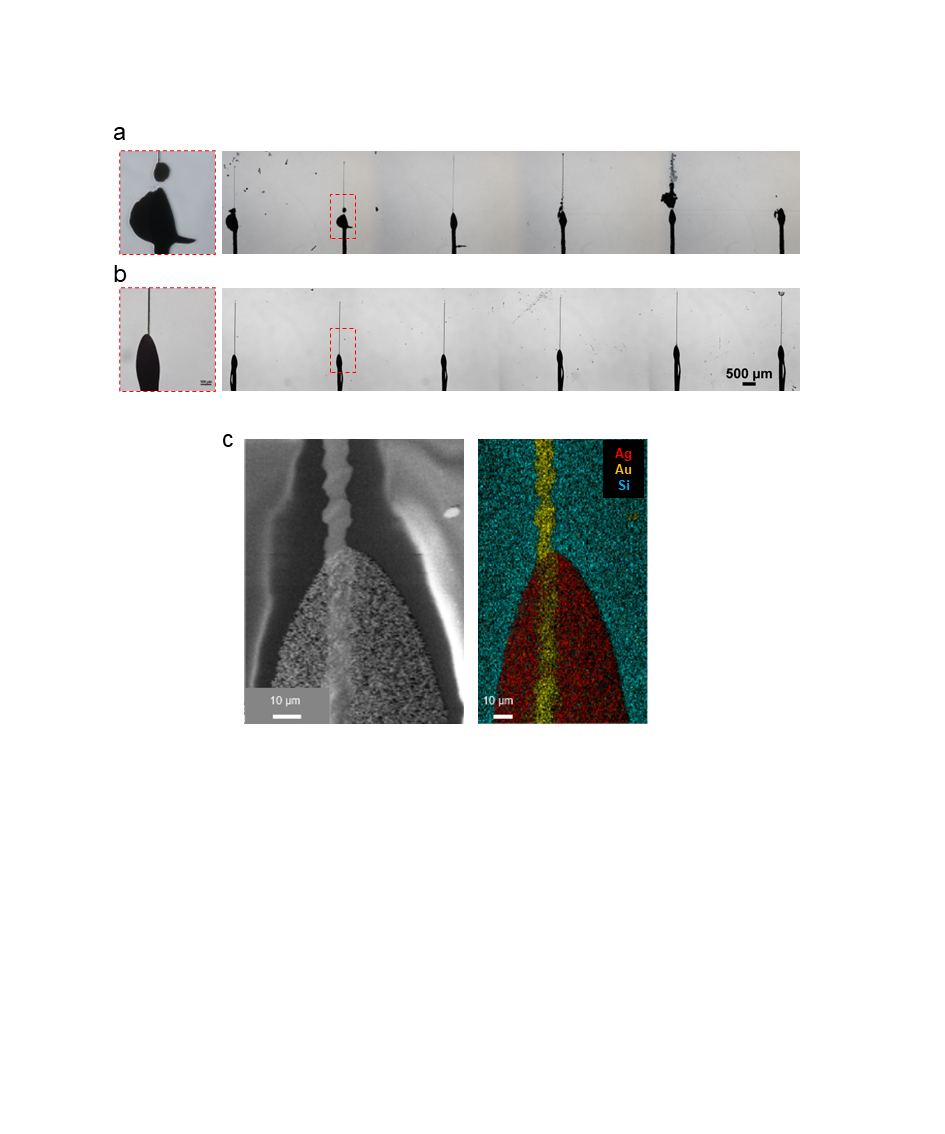


Figure S3. Effect of the laser on EHD-printing the connected silver and gold lines. (a) Optical images show the printed silver and gold lines without the use of the laser. The EHD-printed gold line tends to break with the preprinted silver line at the connection. (b) Optical images show the printed silver and gold lines with the use of the laser. The EHD-printed gold lines are well-connected with the preprinted silver lines. (c) SEM image and corresponding spectral scanning image show the connection of the silver-gold line printed on a glass substrate.


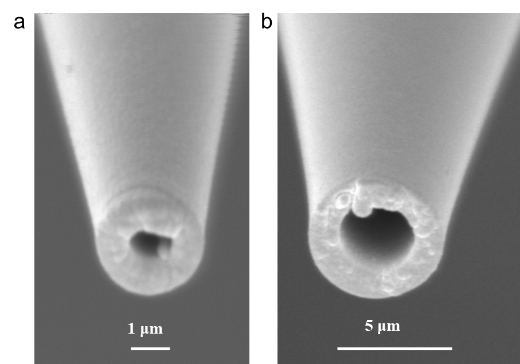


Figure S4. Characterization of the printing nozzles. (a) Scanning electron microscope (SEM) image shown the tip of a glass capillaries with small inner diameters to printing the sub-micron scale 3D gold pillars. (b) SEM image shown the tip of a glass capillaries with large inner diameters to print gold bioelectrodes.


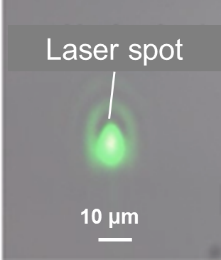


Figure S5. Optical image shows the focused 532 nm laser spot.

**
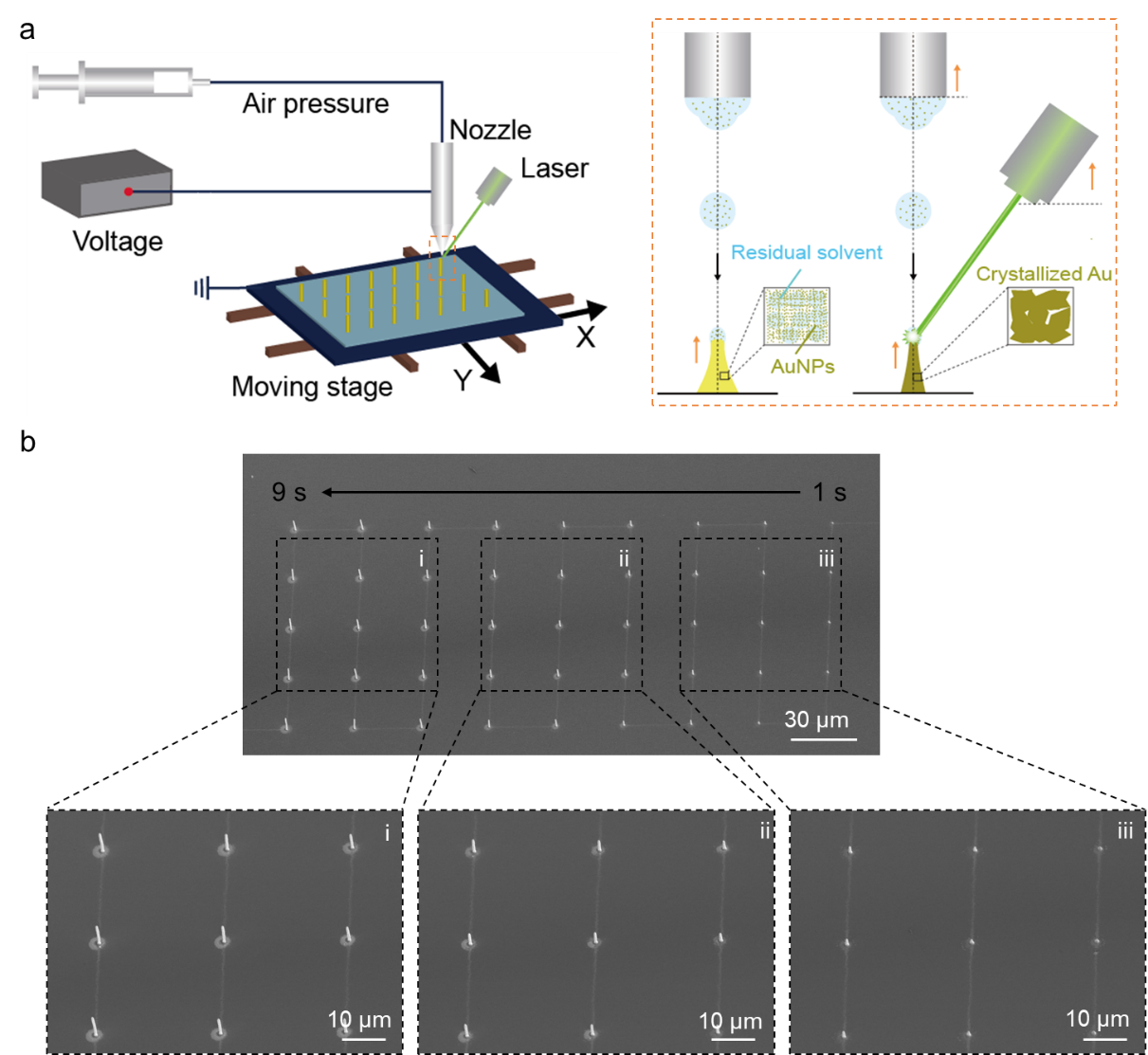
**

Figure S6. Laser-assisted EHDP of 3D gold pillars using the diluted dispersion of gold nanoparticles. (a) Schematic and illustrates the laser-assisted EHDP process of the 3D gold pillars. The inset illustrates the laser-induced solvent evaporation and local sintering of gold nanoparticles. (b) SEM image illustrates the printed 3D gold pillars with printing time from 1s to 9s. Enlarged SEM views show the structure of the printed 3D gold pillars corresponding to the dashed areas.

\


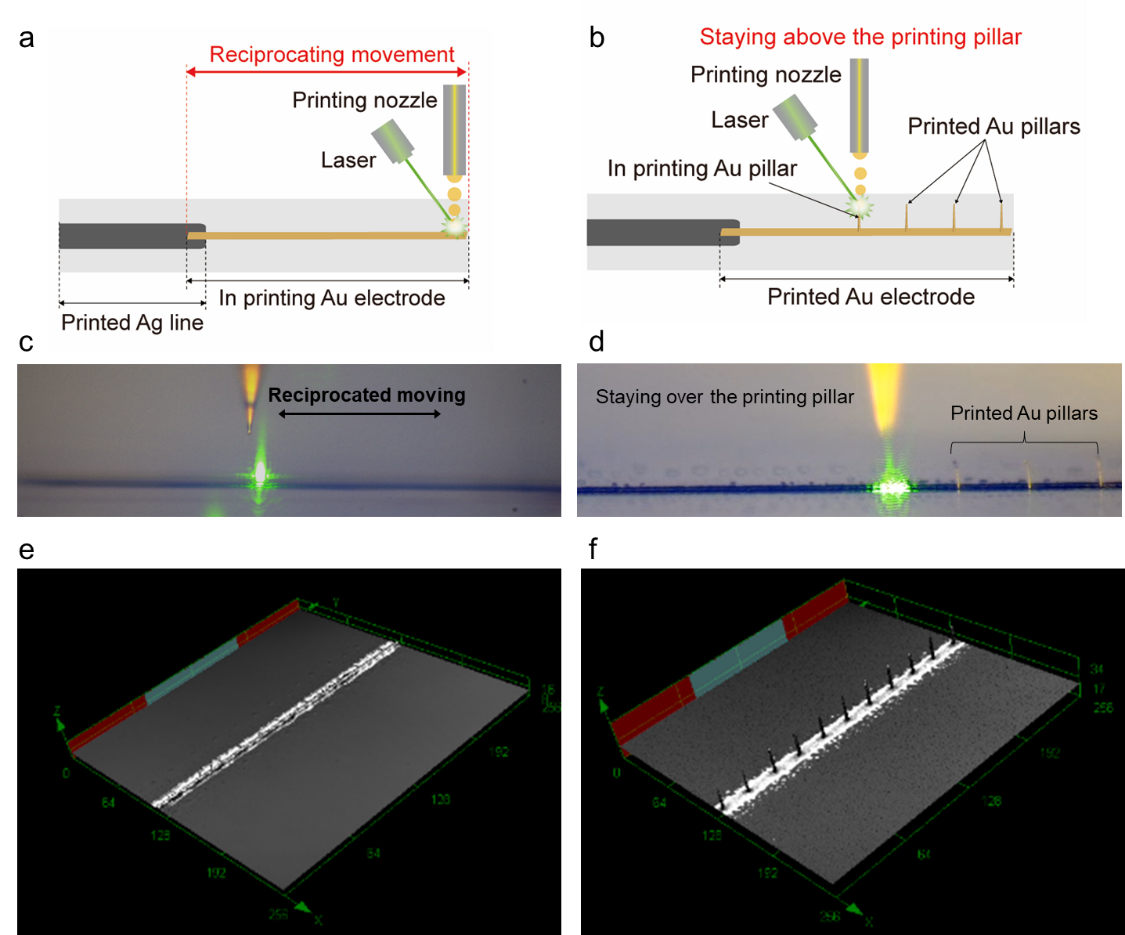


Figure S7. Laser-assisted EHDP of the gold electrode and 3D gold. (a) Schematics show the printing process of the gold electrode and (b) the 3D gold pillars. (c) Optical images show the printing process of the gold electrode and (d) the 3D gold pillars. (e) Laser confocal scanning images show the printed gold electrode and (f) the gold electrode with an array of 3D gold pillars.


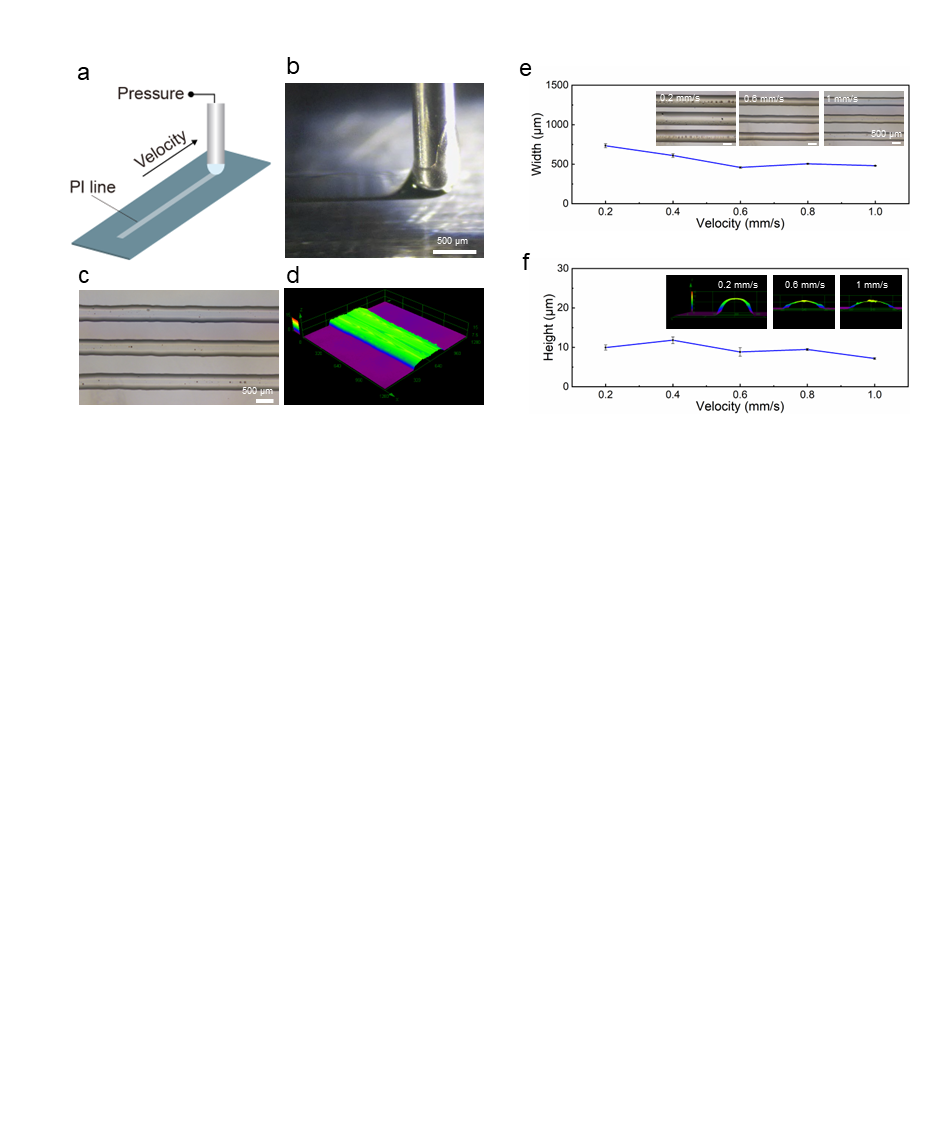


Figure S8. Extrusion-based printing of polyimide lines using the polyimide solution. (a) Schematic and (b) photograph illustrates the printing process of the polyimide line. (c) Optical image and (d) laser confocal image shows the structure of the printed polyimide line. (e) The relationship between the moving velocity of the printing nozzle and the width of the printed polyimide lines. (f) The relationship between the moving velocity of the printing nozzle and the height of the printed polyimide lines.


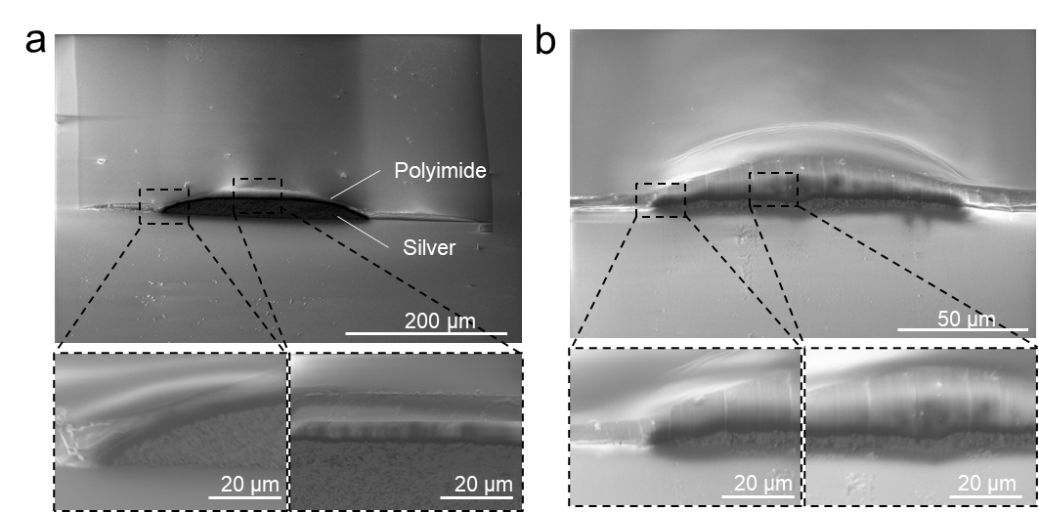


Figure S9. SEM images illustrate the cross-section of overlapping silver and polyimide structures. The inner silver tracks exhibit (a) approximately 200 μm in and (b) approximately 100 μm in width were all uniformly and completely covered by the polyimide layer.

The capacitance (C) of the printed bioelectrode was calculated using the following equation:

$\boldsymbol{C}\boldsymbol{=}\frac{\int_{\boldsymbol{V}_{\boldsymbol{1}}}^{\boldsymbol{V}_{\boldsymbol{2}}} \boldsymbol{I}_{\boldsymbol{(V)}}\boldsymbol{dV}}{\boldsymbol{k}}$ (Equation S1)

,where $\int_{\boldsymbol{V}_{\boldsymbol{1}}}^{\boldsymbol{V}_{\boldsymbol{2}}} \boldsymbol{I}_{\boldsymbol{(V)}}\boldsymbol{dV}$ is the surface area of the plotted cyclic voltammetry (CV) curve, ***k*** is the scan rate (100 mV/s), ***V2-V1*** is the voltage range, ***V1*** is 0.5 V and ***V2*** is 1.1 V.


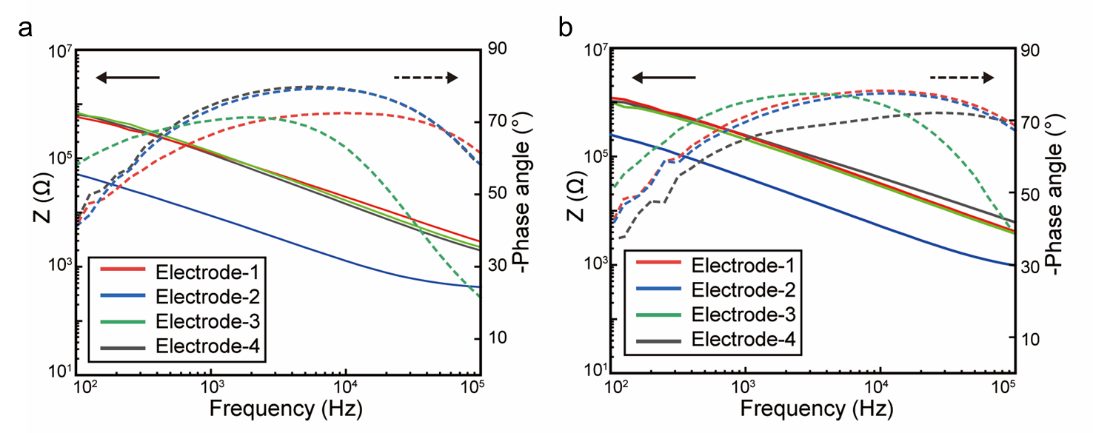


Figure S10. Electrochemical impedance and phase angle spectroscopy of the printed bioelectrodes (a) with 3D pillars and (b) without 3D pillars. Impedance magnitude and phase angle of the impedance spectrum over the frequency range of 10^2^ and 10^5^ Hz.


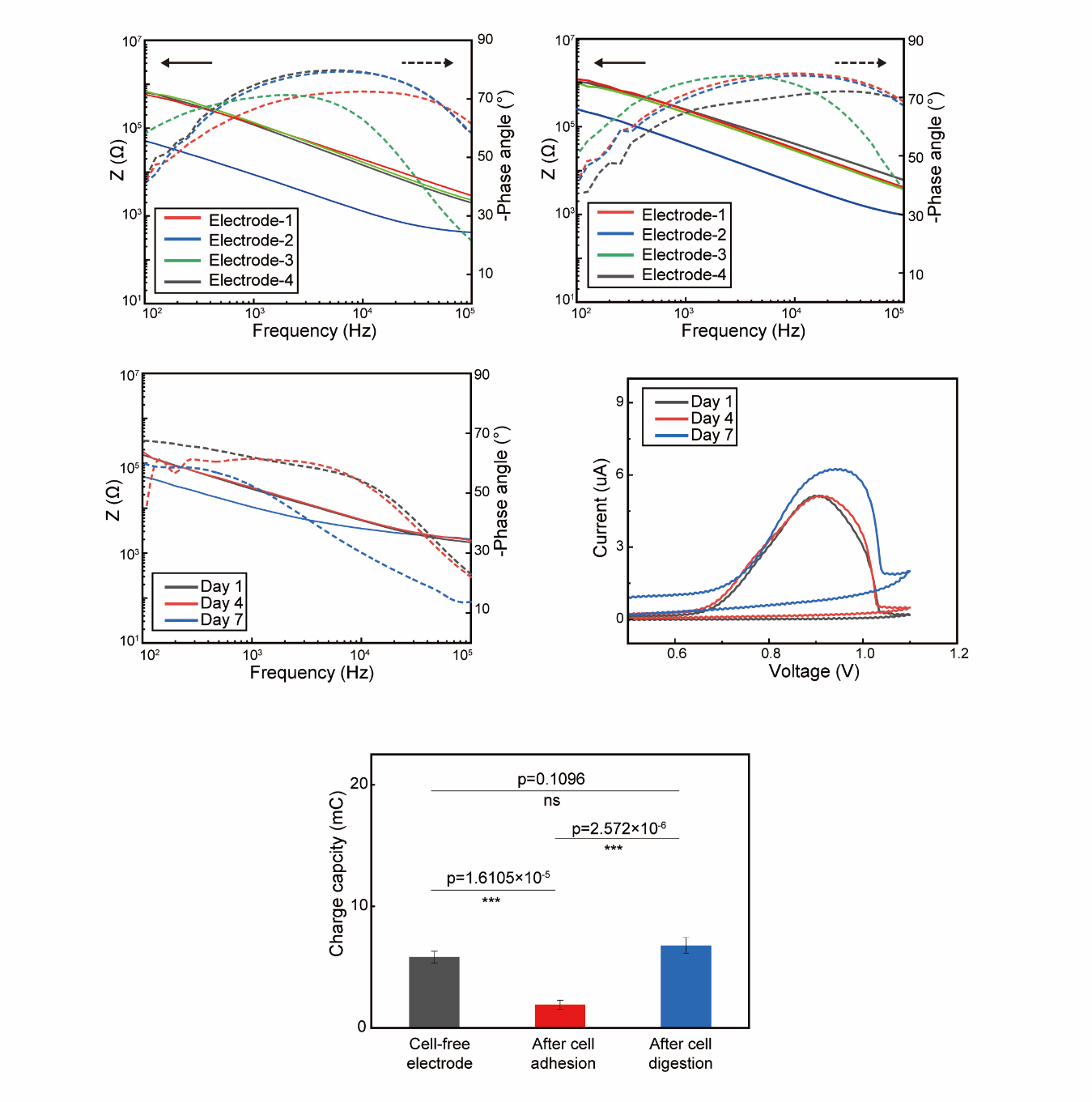


Figure S11. Impedance and phase angle spectrum of the printed bioelectronics after being placed in the 37 ℃ cell incubator for 1, 4 and 7 days. over the frequency range of 10^2^ and 10^5^ Hz.


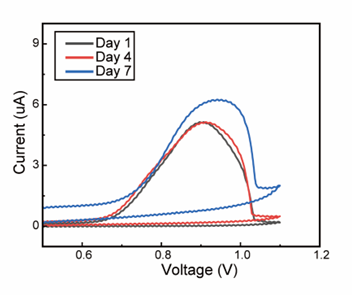


Figure S12. CV curves of the printed bioelectronics after being placed in the 37 ℃ cell incubator for 1, 4 and 7 days.


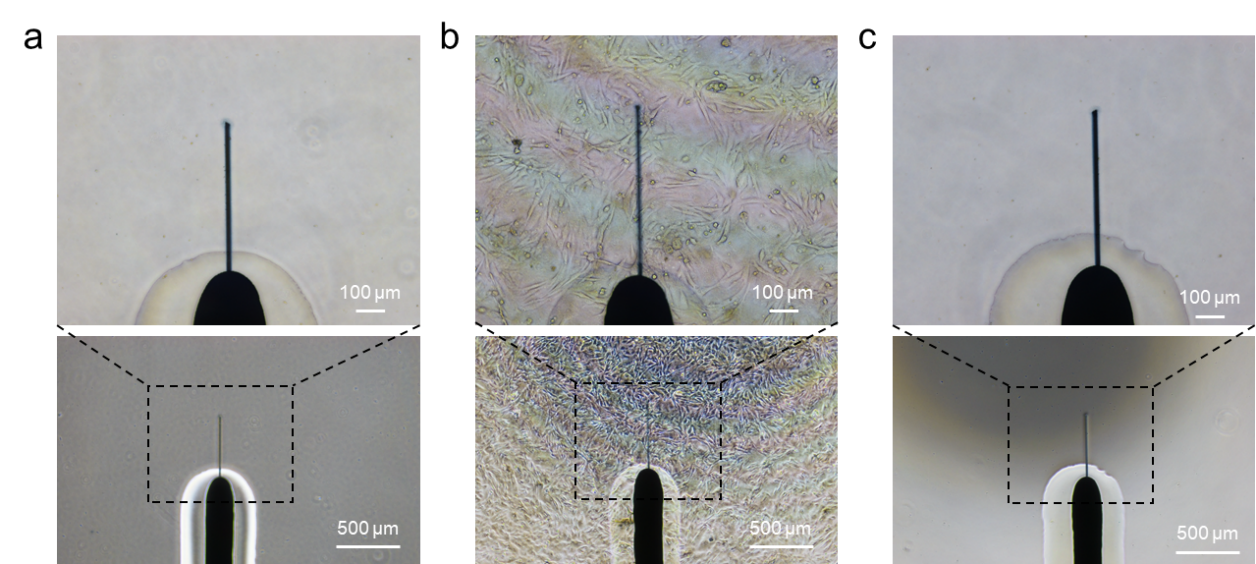


Figure S13. Optical images of the bioelectrode (a) before cell culture, (b) cultured with a confluent layer of HL-1 cells, (c) after cell culture.


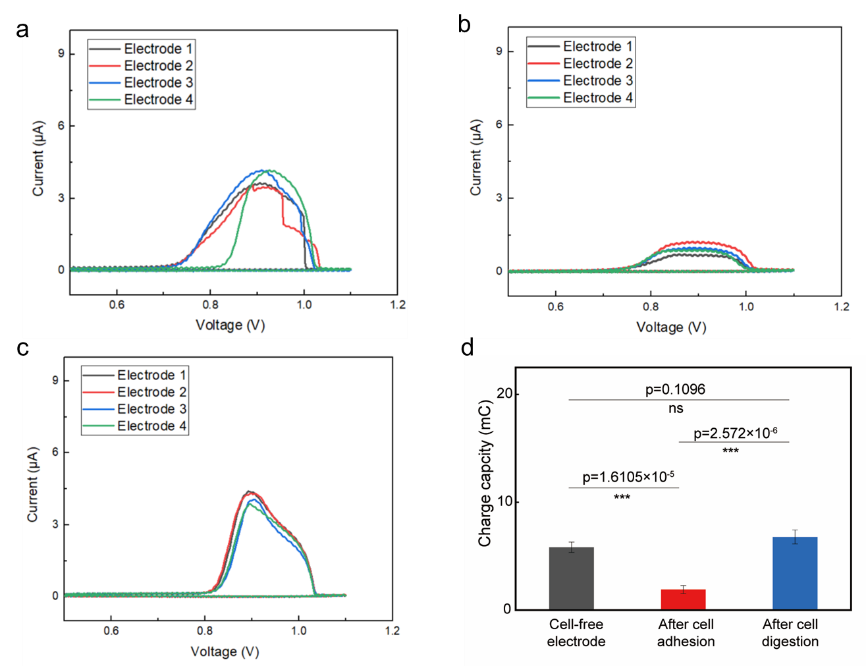


Figure S14. CV curves of (a) the cell-free bioelectrodes, (b) bioelectrodes cultured with a confluent layer of HL-1 cells, (c) bioelectrodes after cell culture, and (d) shows the average charge capacity of bioelectrodes in the three different states, which was calculated according to the plotted CV curves in (a-c).


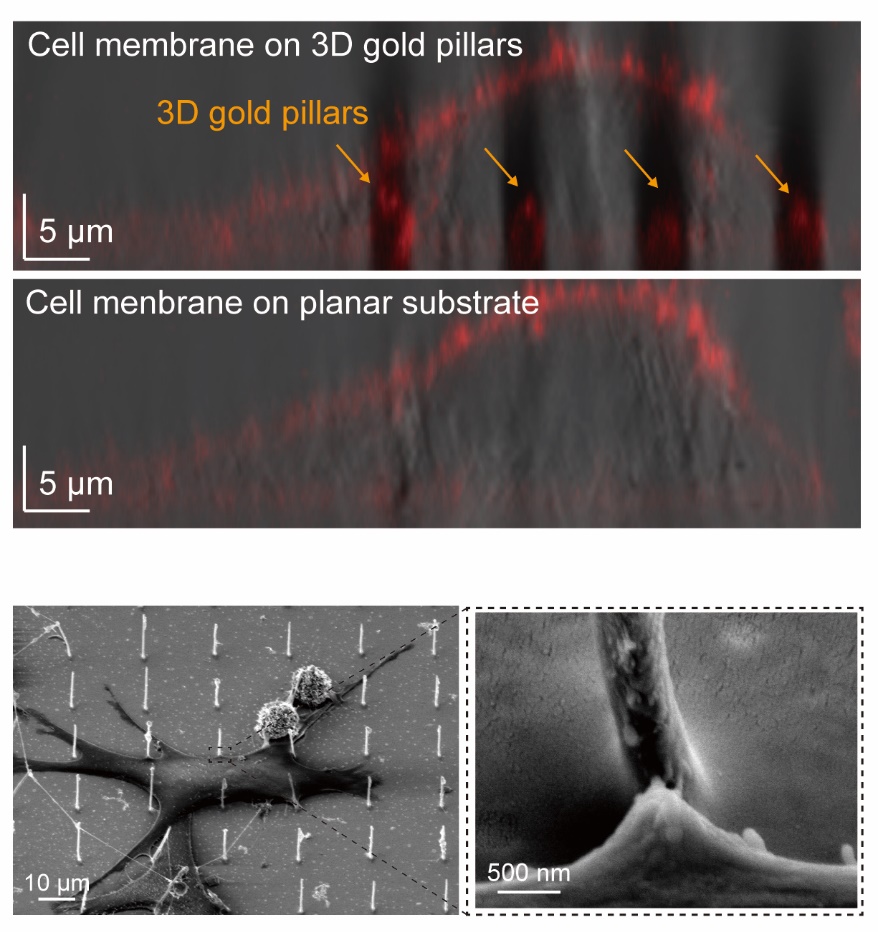


Figure S15. SEM images illustrate the interface between HL-1 cells and the printed 3D gold structures with approximately 10 μm in height.


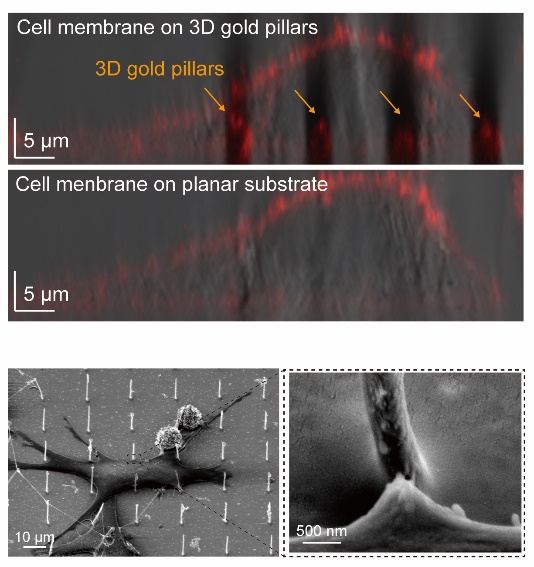


Figure S16. Fluorescent images show the local cell membrane engulfing on the printed 3D gold pillars and attaching to the flat substrate.


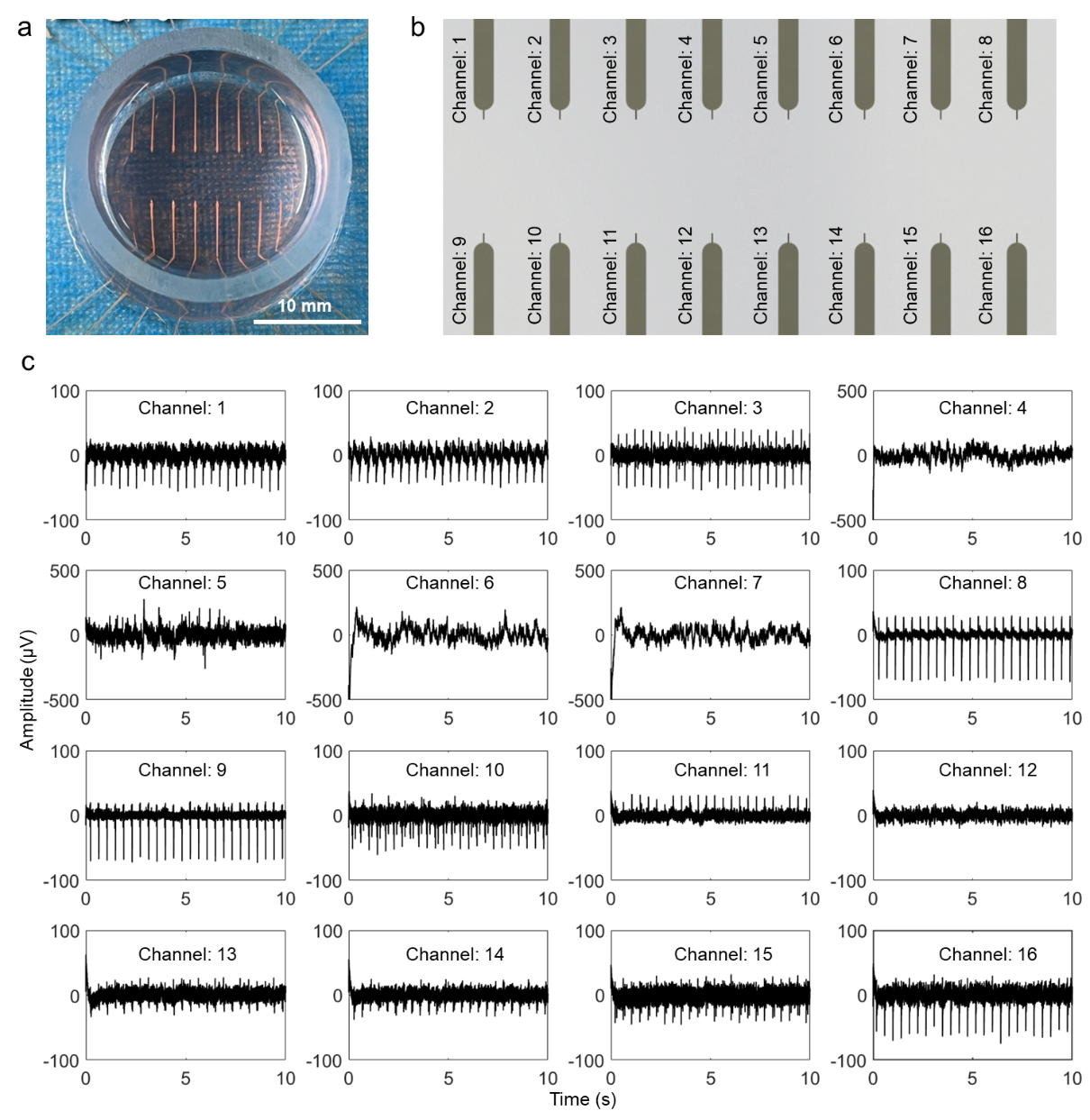
Figure S17. A map of 16-channel signals recorded from the same chip. (a) An optical image shows the printed bioelectronics with 16 independently addressable channels. (b) A schematic illustrates the channel number of the 16-channel bioelectronics. (c) A 4 by 4 map shows the recorded signals from the 16 channels, the channel 1, 2, 3 and 8 to 16 exhibited distinguishable electrophysiological signal spikes.


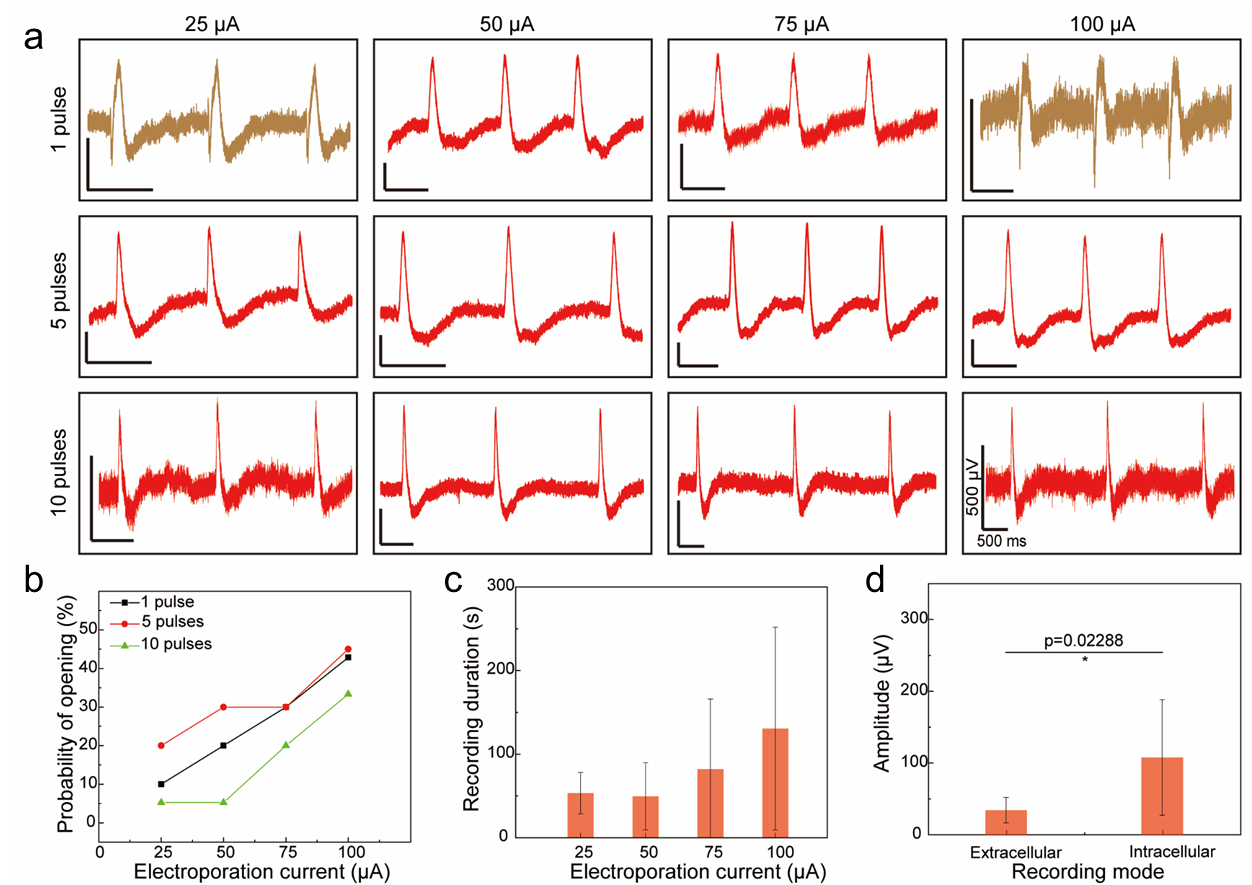


Figure S18. Effects of electroporation pulse parameters on the recorded intracellular signals. (a) Intra-cellular signal traces recorded following different electroporation currents. (b) Probability of inducing intra-cellular recording versus the pulse number and amplitude of the applied electroporation currents. (c) The average duration of intra-cellular recording versus the amplitude of the electroporation currents (data: mean ± SD, n≥3). (d) Enhancement of the signal amplitude from the extracellular to intracellular recording mode using the selected parameters (data: mean ± SD, n=9, p=0.02288).

The quality of intra-cellular recording using printed bioelectronics with sub-microscale 3D gold pillars highly depends on the parameters of the electroporation pulse [1]. To determine the suitable electroporation current pulse sequence, a single pulse, 5 or 10 trains of pulses with amplitudes of 25/50/75/100 μA were induced to the bioelectrode with sub-microscale 3D gold pillars. **Figure S18**(a) displays typical signals recorded right after the application of the different electroporation pulses. Notably, variations in the shape and amplitude of the recorded intra-cellular signals existed. Specifically, the shape of the signal recorded after the single pulse with an amplitude of 25 and 100 μA showed a sharp spike followed by a triangular peak, exhibiting the superposition of intra-cellular and extra-cellular signals, which can be explained by insufficient membrane poration. The signal recorded after the 5 or 10 trains of pulses with all amplitudes showed distinct triangular peaks, demonstrating that the electroporation pulse with adequate number was more likely to induce the intra-cellular recording state. In addition, the variation in amplitude among the signals recorded on different electroporation conditions was observed, which can be attributed to the differences in the coupling strength of the cell-bioelectrode interface [2]. To quantitatively analyze the influence of the electroporation current on the signal recording states, the probability of inducing the intra-cellular recording state and the sustainability of the intra-cellular recording state were characterized. As shown in Figure S18(b), the probability of inducing the intra-cellular recording was significantly improved by increasing the amplitude of the electroporation current, and the 5-pulse train with the pulse amplitude of 100 μA exhibited a higher possibility of inducing the intra-cellular recording that reached approximately 45%. Moreover, by applying the 5-pulse train with different pulse amplitudes, the duration time of intra-cellular recording was increased with the increase of the pulse amplitude, which can be sustained for over 2 min by applying the electroporation pulse with the amplitude of 100 μA (Figure S18(c)). In addition, using the 5-pulse train with a pulse amplitude of 100 μA, the amplitude of the induced intracellular signals (with an average amplitude of 107.75 ± 80.56 μV) was significantly increased compared with the extracellular signals (with an average amplitude of 34.37 ± 17.63 μV) before electroporation (Figure S18(d)). As a result, the 5-pulse train with a pulse amplitude of 100 μA was selected to induce the intra-cellular recording in the following experiments.


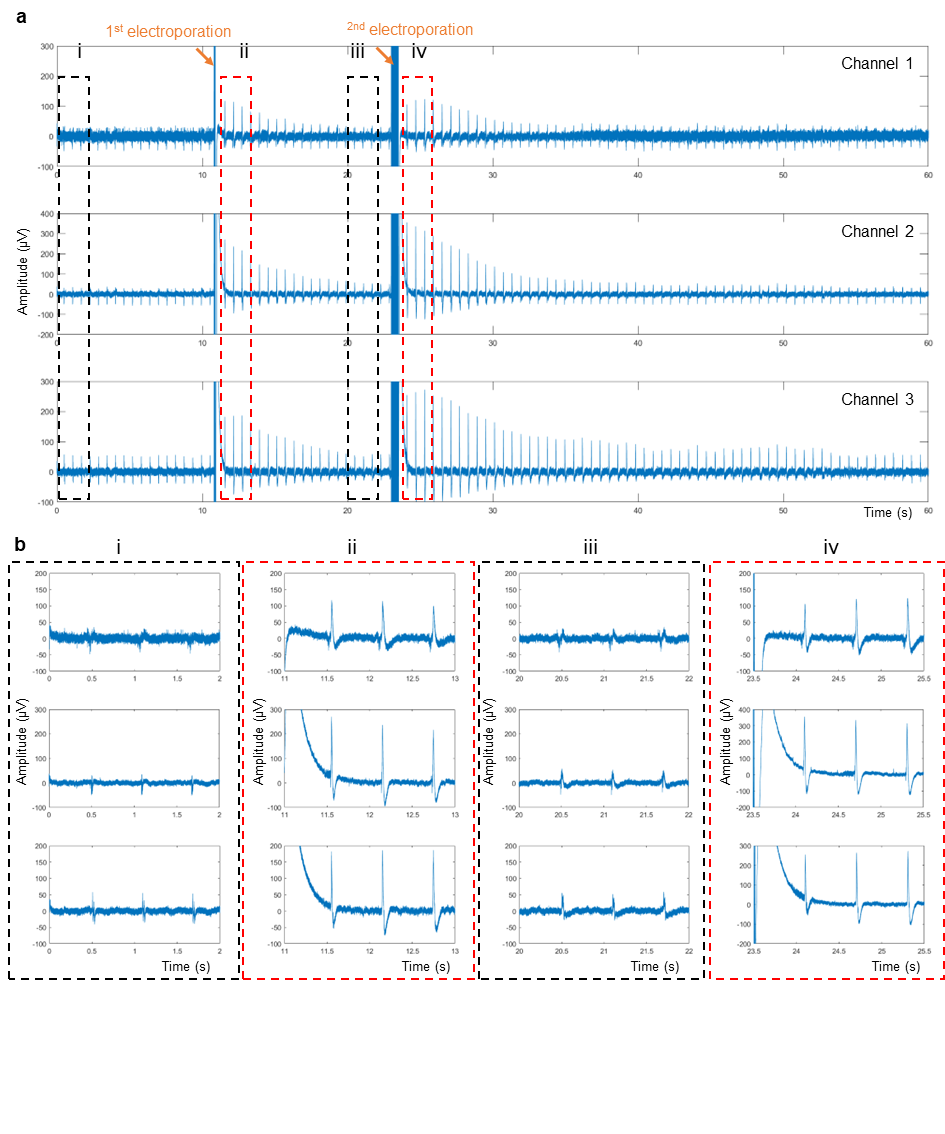


Figure S19. Repeated electroporation and real-time recorded signal traces. (a) Electrophysiological signals were recorded from three channels in real-time, and two times of electroporation were exerted at about 10 and 23 seconds. (b) The enlarged view of the signal trace shows the waveform before electroporation (time window i), after the first electroporation (time window ii), after the signal recovers to the extracellular features (time window ii), and after the second electroporation (time window iv).


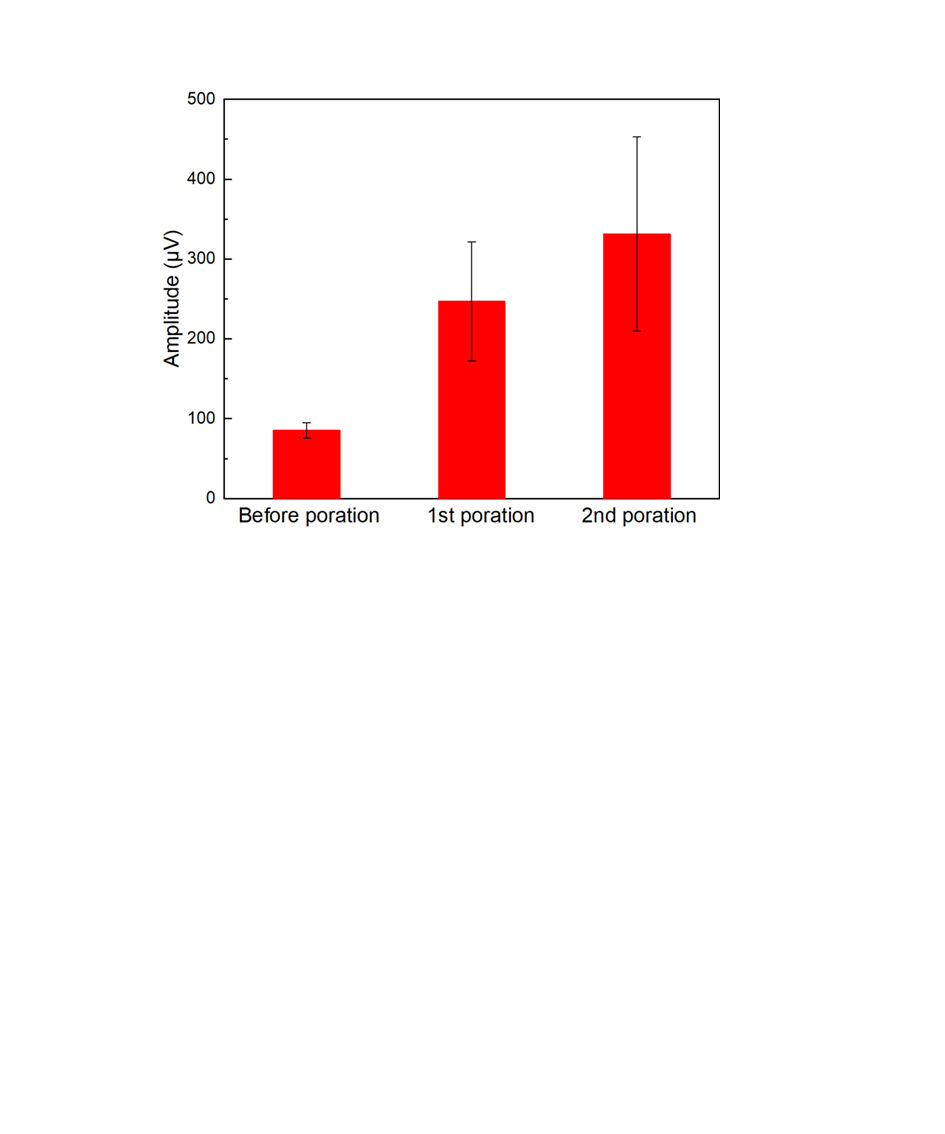


Figure S20. Quantitative comparison of the signal amplitude before electroporation, after the first electroporation, and after the second electroporation.


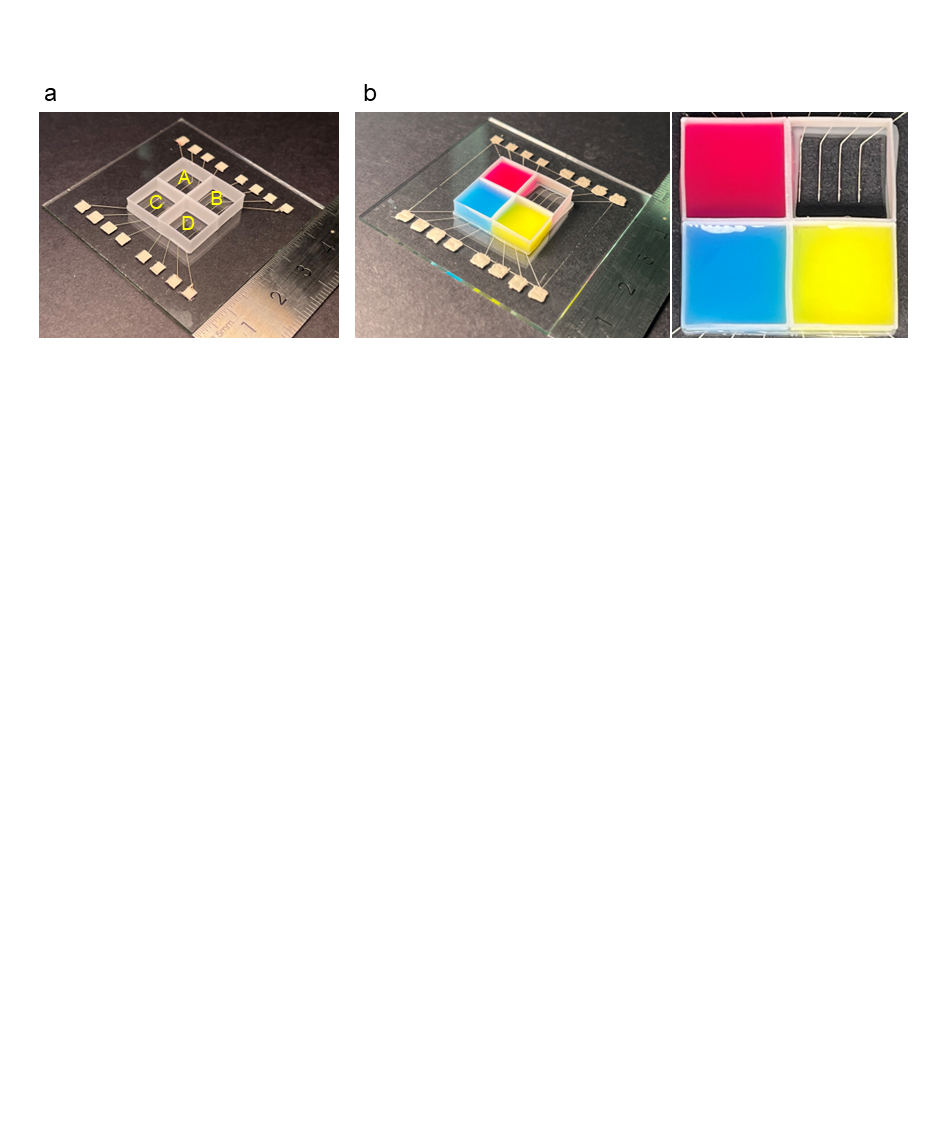


Figure S21. Printed wells using biocompatible thermoplastic polycaprolactone (PCL) polymer. Photographs show the fully-printed bioelectronic device with the PCL wells that divide the 16 electrodes into four sections.

Table S1. Data showing the reduction of frequency as shown in Figure 5e.

| Chambers of  the same chip | Treatment | Spike frequency (Spikes/min) | | |
| --- | --- | --- | --- | --- |
|  |  | Chip1 | Chip2 | Chip3 |
| Chamber1 (0 μM) | Before drug | 100 | 128 | 131 |
|  | After drug | 100 | 128 | 131 |
| Chamber2 (0.5 μM) | Before drug | 42 | 150 | 114 |
|  | After drug | 31 | 156 | 114 |
| Chamber3 (1 μM) | Before drug | 108 | 101 | 88 |
|  | After drug | 75 | 29 | 68 |
| Chamber4 (5 μM) | Before drug | 114 | 128 | 156 |
|  | After drug | 20 | 73 | 89 |

## References

[1] Xu D, Fang J, Yadid M, Zhang M, Wang H, Xia Q, et al. A universal, multimodal cell-based biosensing platform for optimal intracellular action potential recording. Biosensors & Bioelectronics. 2022;206.

[2] Dipalo M, McGuire AF, Lou H-Y, Caprettini V, Melle G, Bruno G, et al. Cells Adhering to 3D Vertical Nanostructures: Cell Membrane Reshaping without Stable Internalization. Nano Letters. 2018;18:6100-5.
